# Effect of present state bias on minimal important change estimates: a simulation study

**DOI:** 10.1007/s11136-024-03763-4

**Published:** 2024-08-22

**Authors:** Berend Terluin, Piper Fromy, Andrew Trigg, Caroline B. Terwee, Jakob B. Bjorner

**Affiliations:** 1 Department of General Practice, Amsterdam UMC, Vrije Universiteit Amsterdam, de Boelelaan 1117, 1081 HV Amsterdam, The Netherlands; 2 Amsterdam Public Health Research Institute, Amsterdam, The Netherlands; 3 SeeingTheta, 2 Chemin des Vaux, 49400 Saumur, France; 4 Medical Affairs Statistics, Bayer Plc, Reading, UK; 5 Department of Epidemiology and Data Science, Amsterdam UMC, Vrije Universiteit Amsterdam, Meibergdreef 9, 1105 AZ Amsterdam, The Netherlands; 6 QualityMetric, Johnston, RI USA; 7 Department of Public Health, University of Copenhagen, Copenhagen, Denmark; 8 National Research Centre for the Working Environment, Copenhagen, Denmark

**Keywords:** Minimal important change, Meaningful change threshold, Present state bias, Transition ratings, Patient-reported outcome measure

## Abstract

**Purpose:**

The minimal important change (MIC) in a patient-reported outcome measure is often estimated using patient-reported transition ratings as anchor. However, transition ratings are often more heavily weighted by the follow-up state than by the baseline state, a phenomenon known as “present state bias” (PSB). It is unknown if and how PSB affects the estimation of MICs using various methods.

**Methods:**

We simulated 3240 samples in which the true MIC was simulated as the mean of individual MICs, and PSB was created by basing transition ratings on a “weighted change”, differentially weighting baseline and follow-up states. In each sample we estimated MICs based on the following methods: mean change (MC), receiver operating characteristic (ROC) analysis, predictive modeling (PM), adjusted predictive modeling (APM), longitudinal item response theory (LIRT), and longitudinal confirmatory factor analysis (LCFA). The latter two MICs were estimated with and without constraints on the transition item slope parameters (LIRT) or factor loadings (LCFA).

**Results:**

PSB did not affect MIC estimates based on MC, ROC, and PM but these methods were biased by other factors. PSB caused imprecision in the MIC estimates based on APM, LIRT and LCFA with constraints, if the degree of PSB was substantial. However, the unconstrained LIRT- and LCFA-based MICs recovered the true MIC without bias and with high precision, independent of the degree of PSB.

**Conclusion:**

We recommend the unconstrained LIRT- and LCFA-based MIC methods to estimate anchor-based MICs, irrespective of the degree of PSB. The APM-method is a feasible alternative if PSB is limited.

**Supplementary Information:**

The online version contains supplementary material available at 10.1007/s11136-024-03763-4.

## Introduction

In the evaluation of clinical research and practice, patient-reported outcomes (PROs), such as pain or physical function, have acquired an increasingly important role [1]. Typically, questionnaires, referred to as patient-reported outcome measures (PROMs), are used to measure such PROs at multiple time points, to assess change. Because PROM change scores lack intrinsic meaning, the concept of minimal important change [MIC; also called minimal (clinically) important difference (MCID or MID), meaningful within‑individual change (MWIC), or meaningful change threshold (MCT)], was introduced to help researchers and clinicians to interpret the clinical significance of PROM change scores [2]. The MIC is the smallest within-individual PROM change score that patients consider meaningful or important. Given the assumption that patients have their own MIC thresholds, the MIC-value to be estimated in a group of patients can be conceptualized as the mean of individual MICs [3–5].^1^

Anchor-based methods to estimate an MIC use an external criterion (i.e., the anchor) as a measure of minimal importance. A frequently used anchor is the transition (or global impression of change) rating provided by patients at follow-up. A typical transition question is “Please choose the response that best describes the overall change in your <symptom/overall status/etc.> since you started taking the study medication. Much worse, A little worse, No change, A little better, Much better” [6].

Using a transition question as an anchor assumes that patients are able to assess their change between a previous state (i.e., the baseline state) and the present state (i.e., the follow-up state). This requires that patients remember their previous experience to compare it with their present experience. However, with the passage of time, human memory is fallible. The resulting “noise” can lead to transition ratings that are relatively stronger determined by the present state than by the previous state [7, 8]. This so-called “present state bias” (PSB) is, to a variable extent, likely to be common. Recently, a method was introduced to accurately estimate the degree of PSB in transition ratings [9]. Now that we are able to estimate the degree of PSB in real data, the question arises to what extent PSB affects MIC estimation. Therefore, we conducted a simulation study to assess the effect of PSB on anchor-based MICs, estimated using various methods.

## Methods

### Data simulation

#### PROM item response data

We used item response theory (IRT) to simulate multiple datasets with item responses at two time points, baseline (T1) and follow-up (T2). IRT entails a set of mathematical models describing the relationship between responses to an item of a PROM and the underlying unobserved construct (or “latent trait”, denoted by the Greek letter “theta”, $\theta$) that the PROM purports to measure [10]. “Theta” is also used to indicate the latent trait metric (or scale), typically with a mean of 0 and a standard deviation (SD) of 1 in estimated IRT-models. We used the graded response model (GRM) for polytomous items [11]. The GRM describes the probability of endorsing a particular item response category (or a higher response category of that item) as a function of the latent trait $\theta$ and two item parameters, “location” and “slope”. The location parameter refers to the location on the latent trait where the probability of endorsing an item response category (or a higher category) is 50%, and the slope parameter refers to the slope of the item response function curve showing the probability of endorsing an item response option as a function of the underlying latent trait.

We simulated complete sets of item responses for 2000 “simulees” (i.e., simulated patients), based on a set of item parameters and simulated distributions of the latent traits at baseline (${\theta }_{\text{T}1}$) and follow-up (${\theta }_{\text{T}2}$). The difference between ${\theta }_{\text{T}1}$ and ${\theta }_{\text{T}2}$ is denoted “latent change” (i.e., $\Delta \theta ={\theta }_{\text{T}2}-{\theta }_{\text{T}1}$). We created a set of item parameters for ten items with four response options^2^ (see the Online Resource, Sect. 1). The latent trait and change variables were created as follows. For each simulated dataset, we created a latent trait variable for T1 (${\theta }_{\text{T}1}$) and a latent change variable ($\Delta \theta$) that was or was not correlated with ${\theta }_{\text{T}1}$. The latent trait variable at T2 (${\theta }_{\text{T}2}$) was then calculated by adding the $\Delta \theta$ values to the ${\theta }_{\text{T}1}$ values.

The latent variable at T1 (${\theta }_{\text{T}1}$) was given an SD of 1 and a mean of − 1, 0 or 1, simulating baseline samples with relatively low, average or high mean levels of the trait.^3^ The latent change variable ($\Delta \theta$) was given an SD of 0.75, 1 or 1.25, simulating less or more variability in the latent change. The correlation between ${\theta }_{\text{T}1}$ and $\Delta \theta$ was set at − 0.50 or 0. The mean of the latent change ($\overline{\Delta \theta }$) was chosen in such a way that the proportion improved (i.e., the proportion of simulees whose latent change exceeded their individual MIC—see below) was 0.2, 0.5 or 0.8.

#### Transition ratings

In the absence of PSB, transition ratings with 5 response options (1 = “Much worse”, 2 = “A little worse”, 3 = “No change”, 4 = “A little better”, 5 = “Much better”) were simulated as follows. First, we simulated 4 variables with means of − 1.5, − 0.5, 0.5 and 1.5 and an SD of 0.075, representing the thresholds between the transition response options on the $\Delta \theta$ scale. The 3rd threshold (between “No change” and “A little better”) was considered to represent the individual MIC, and the mean value represented the MIC (in the $\Delta \theta$ metric) to be estimated. Second, in each dataset, we simulated a “perceived change” variable by adding a random error variable to $\Delta \theta$. The mean of the error variable was 0 and its SD was chosen as to create a reliability of the perceived change of 0.3 or 0.5.^4^ Then, the transition rating responses were derived by comparing each simulee’s perceived change with their individual thresholds. Simulees whose perceived change did not exceed the first threshold were given code “1” (“Much worse”), simulees whose perceived change exceeded the first but not the second threshold were given code “2” (“A little worse”), and so on. It should be noted that the reliability of the transition ratings, being a discretization of the perceived change, equaled the reliability of the perceived change (i.e., 0.3 or 0.5).

#### Present state bias

In the presence of PSB, transition ratings were simulated by creating a “weighted (latent) change” variable (${\Delta \theta }_{w}$) to replace the (unweighted) latent change ($\Delta \theta$) in the procedure to obtain transition ratings as described above. Whereas the *unweighted* latent change was $\Delta \theta ={\theta }_{\text{T}2}-{\theta }_{\text{T}1}$, the following formula was used for the *weighted* latent change:

1 $$\begin{aligned} \Delta \theta_w & = q \times \left( {\theta_{{\text{T}}2} - \overline{{\theta_{{\text{T}}1} }}} \right) + \left( {1 - q} \right) \times \left( {\theta_{{\text{T}}2} - \theta_{{\text{T}}1} } \right)\quad {\text{or, equivalently:}} \\ \Delta \theta_w & = q \times \left( {\theta_{{\text{T}}2} - \overline{{\theta_{{\text{T}}1} }}} \right) + \left( {1 - q} \right) \times \Delta \theta \\ \end{aligned}$$

where $q$ represents a variable of weights between 0 and 1, indicating the degree of PSB (0 = no PSB; 1 = complete PSB), and where $\overline{{\theta }_{\text{T}1}}$ is the mean of the baseline state (${\theta }_{\text{T}1}$). Equation (1) indicates that ${\Delta \theta }_{w}$ consists of two components: a proportion $q$ of the latent follow-up state (i.e., the present state) relative to the mean baseline state (i.e., ${\theta }_{\text{T}2}-\overline{{\theta }_{\text{T}1}}$), and a complementary proportion $(1-q)$ of the true latent change ($\Delta \theta$, i.e., ${\theta }_{\text{T}2}-{\theta }_{\text{T}1}$). The mean of the baseline state ($\overline{{\theta }_{\text{T}1}}$) is included in the Equation for the following reason. Consider patients whose transition ratings show complete PSB and who, therefore, for rating their transition, solely rely on their present state (${\theta }_{\text{T}2}$) for comparison with their individual thresholds. Such patients “treat” their present state (${\theta }_{\text{T}2}$) as if it were their true change. However, because ${\theta }_{\text{T}2}$ itself represents a state (i.e., the end result of change) and not a change, evaluating ${\theta }_{\text{T}2}$ as if it represented change requires that people construct a reference point for their baseline state. This reference point construction is probably a vague, implicit and intuitive process. It is assumably not more than a (wild) guess about where they were at baseline, probably more or less below or above the point where they truly were at that time. If the latter assumption is true, i.e., if people are as likely to underestimate as they are to overestimate their baseline state, we may assume that the mean of the constructed individual reference points equals the mean of the true baseline state (i.e., $\overline{{\theta }_{\text{T}1}}$). This might be seen as an example of “wisdom of the crowd”, the phenomenon that the average of many individual estimates tends to approximate the truth [13].^5^

We simulated $q$-values as a variable with a mean and an SD, accounting for the idea that the degree of PSB probably varies across individual people. The mean $q$-values were varied between 0 and 1 in steps of 0.2. To obtain, for instance, a weighted change variable with a group-level (mean) PSB of 40%, variable $q$ was simulated as a random variable with a mean of 0.4 and an SD of (0.5 − |(0.5 − 0.4)|)/4.^6^ Individual $q$-values < 0 or > 1 were constrained to 0 and 1 respectively. In this example, 40% PSB implies that the average weighted latent change consisted of 40% of $({\theta }_{\text{T}2}-\overline{{\theta }_{\text{T}1}})$ and 60% of $\Delta \theta$. Once ${\Delta \theta }_{w}$ was created, error was added to obtain the “perceived change” variable with the desired reliability values (0.3 or 0.5), and, subsequently, the transition ratings were created, as described above.

Table 1 summarizes the parameters that were varied across the simulated datasets. There were 648 unique combinations of parameters. Each combination was simulated five times, resulting in a total of 3240 simulated samples. Descriptive sample characteristics are summarized in Table 2.

**Table 1:** Parameters varying across the simulated samples

Parameter	Values	Explanation
Mean latent trait T1 ($\overline{{\theta }_{\text{T}1}}$)	− 1, 0, 1	$\overline{{\theta }_{\text{T}1}}$ reflects the average level of the latent trait at T1
SD latent change (SD $\Delta \theta$)	0.75, 1, 1.25	SD $\Delta \theta$ reflects the variability in the latent change between T1 and T2 ($\Delta \theta$)
Correlation between ${\theta }_{\text{T}1}$ and $\Delta \theta$	− 0.50, 0.00	Values are correlation coefficients
Proportion improved	0.2, 0.5, 0.8	Proportion of simulees having a true latent change greater than their individual MIC
Reliability of the transition ratings	0.3, 0.5	Values are reliability coefficients
Present state bias	0, 0.2, 0.4, 0.6, 0.8, 1	Proportion of ${\theta }_{\text{T}2}-\overline{{\theta }_{\text{T}1}}$ that is included in the weighted latent change (${\Delta \theta }_{w}$)

SD, Standard deviation; T1, Baseline; T2, Follow-up

**Table 2:** Sample characteristics across 3240 simulated samples

Characteristic	Mean (range)
Reliability^a^ (T1)	0.86 (0.84, 0.88)
Mean T1 scale score	15.0 (8.4, 21.6)
SD T1 scale score	6.7 (6.2, 7.4)
Skewness T1 scale score	0.01 (− 0.83, 0.85)
Kurtosis T1 scale score	− 0.37 (− 0.97, 0.17)
Floor effects T1 scale score	0.02 (0.00, 0.07)
Ceiling effects T1 scale score	0.02 (0.00, 0.08)
Mean T2 scale score	17.7 (5.7, 27.6)
SD T2 scale score	7.0 (3.4, 9.5)
Skewness T2 scale score	− 0.39 (− 2.45, 1.29)
Kurtosis T2 scale score	0.13 (− 1.22, 7.42)
Floor effects T2 scale score	0.03 (0.00, 0.24)
Ceiling effects T2 scale score	0.09 (0.00, 0.44)
Mean scale change score	2.7 (− 4.2, 10.3)
SD scale change score	6.8 (4.8, 9.1)
Skewness scale change score	0.04 (− 0.44, 0.69)
Kurtosis scale change score	0.13 (− 0.69, 1.24)
Floor effects scale change score	0.00 (0.00, 0.00)
Ceiling effects scale change score	0.00 (0.00, 0.00)

SD, Standard deviation; T1, Baseline; T2, Follow-up

^a^Cronbach’s alpha

### Analysis

#### MIC methods

##### Mean change (MC) method

The MC method is the oldest anchor-based MIC method [2, 14]. It takes the mean change score of the minimally importantly changed subgroup as the MIC. In each simulated sample we calculated PROM scores (i.e., the sum of 10 item scores) at T1 and T2, and the PROM change score (T2 − T1). Then, we recorded the mean change score for the subgroup scoring “A little better” (transition rating “4”) as the mean change MIC (MIC_MC_).

##### Receiver operating characteristic (ROC) method

The ROC method approaches MIC estimation as a diagnostic problem, seeking to classify improved and not-improved patients with the least amount of misclassification [15–17]. In each simulated sample we performed ROC analysis using the dichotomized transition ratings (improved (codes 4–5) versus not-improved [codes 1–3)] as the “state variable” and the PROM change score as the “test variable” [18, 19]. The optimal cut-off point according to the Youden criterion (i.e., maximization of sensitivity and specificity) was recorded as the ROC-based MIC (MIC_ROC_).

##### Predictive modeling (PM) method

This method is based on logistic regression using the dichotomized transition ratings as the outcome and the PROM change score as the determinant [20]. The change score of interest is the one with a likelihood ratio of 1 (i.e., the change score that is equally likely to occur in the improved group as in the not-improved group). The predictive modeling MIC (MIC_PM_) identifies about the same cut-off point as MIC_ROC_, but MIC_PM_ is much more precise [20].

##### Adjusted predictive modeling (APM) method

Because MIC_PM_ is biased by the proportion improved, an adjustment was developed [4] and later improved [21]. In each sample we estimated the reliability of the transition ratings according to the method described by Griffiths et al. [12], and calculated the adjusted predictive modeling MIC (MIC_APM_).

##### Longitudinal item response theory (LIRT) method

The LIRT-based MIC (MIC_LIRT_) is based on an LIRT model of the PROM items, with the dichotomized transition item serving as an indicator of both time factors [22]. In each sample we performed LIRT analysis with the slope parameters (${{\upalpha }}_{\text{TR}1}$ and ${{\upalpha }}_{\text{TR}2}$) of the transition item being negatively constrained as recommended (i.e., ${{\upalpha }}_{\text{TR}1}=-{{\upalpha }}_{\text{TR}2}$) [22]. MIC_LIRT_ was first estimated on the metric of the latent change, as the location parameter of the transition item on the latent change. MIC_LIRT_ in terms of the PROM change score was then calculated as the mean change in expected PROM score if a large sample (n = 500,000) of simulees, in ${\theta }_{\text{T}1}$ distribution similar to the simulated sample, would all improve by exactly one MIC in terms of the latent change [22]. Because constraining the slope parameters of the transition ratings (TR) causes model misfit in the presence of PSB, we also estimated MIC_LIRT_ after freeing the slope parameters. Here, MIC_LIRT_ was estimated based on ${{\upalpha }}_{\text{TR}2}$.

##### Longitudinal confirmatory factor analysis (LCFA) method

The LCFA-based MIC (MIC_LCFA_) is based on an LCFA model for ordinal indicators with the PROM items at T1 and T2 loading on the latent factors at T1 and T2 respectively, and the dichotomized transition item loading on both time factors [23]. The factor loadings of the transition item (${\uplambda }_{\text{TR}1}$ and ${\uplambda }_{\text{TR}2}$) were negatively constrained (i.e., ${\uplambda }_{\text{TR}1}=-{\uplambda }_{\text{TR}2}$). MIC_LCFA_ was first estimated on the metric of the latent change. MIC_LCFA_ in terms of the PROM change score was then calculated in the same way as for MIC_LIRT_. Because constraining the factor loadings causes model misfit in the presence of PSB, we also estimated MIC_LCFA_ after freeing the TR factor loadings. Here, MIC_LCFA_ was estimated based on ${\uplambda }_{\text{TR}2}$.

#### The effect of PSB on the MIC estimates

The effect of PSB was evaluated by comparing the MIC estimates with the “true” MIC value (as simulated), expressing the effect in terms of bias and mean squared error (MSE). Bias represents the mean difference between the MIC estimates and the true MIC. Bias indicates the tendency to systematically over- or underestimate the true value. MSE represents the mean of the squared differences between the MIC estimates and the true value, and indicates both the bias and the (im)precision of the estimates.

Whereas we aimed to estimate the MIC in the metric of the simulated PROM, we simulated the (true) MIC in the metric of the latent change (the true latent MIC was set to 0.5 theta units). The true MIC in terms of the PROM change score was calculated for each simulated sample as the mean change in expected PROM score if a large sample (n = 500,000) of simulees, similar to the simulated sample, would all improve by exactly one MIC in terms of the latent change. This true MIC was then subtracted from the estimated MICs to obtain MIC residuals. Positive residuals indicated overestimation of the true MIC, whereas negative residuals indicated underestimation of the true MIC. To examine the effect of PSB on the MIC, the MIC residuals were plotted against the degree of PSB, and the MIC residuals were summarized in terms of bias, SD, and MSE.

Furthermore, to explore the effect of PSB on the MIC (residuals), relative to other sample characteristics, we performed multivariate linear regression, regressing the MIC residuals on the simulation parameters (see Table 1) and all of their interactions. Then, backward selection was applied to remove interactions and determinant terms explaining < 2% (adjusted R^2^) of the variance.

### Software

All analyses were performed in the statistical program R, version 4.2.2 [24]. We used the mirt package, version 1.40 [25] to simulate the samples, estimate LIRT-based MICs in terms of the latent change, and to estimate MICs in terms of the PROM change score for the LIRT and LCFA methods. We used the packages pROC, version 1.18.4, for ROC analysis [26], lavaan, version 0.6–16, for LCFA [27], and rsimsum, version 0.11.3, for summarizing the simulation results [28]. The R-code is available in the Online Resource, Sect. 5.

## Results

### True MIC in terms of PROM change score

Across the simulated samples the true MIC (in terms of the PROM change score) varied between 2.56 and 3.41 (mean 3.01, SD 0.29). The true MIC appeared to be determined by the mean ${\theta }_{\text{T}1}$ (as simulated). If the baseline trait level was average (i.e., mean ${\theta }_{\text{T}1}$ = 0), the mean true MIC was 3.33 (SD 0.02), if the baseline trait level was relatively low (i.e., mean ${\theta }_{\text{T}1}$ = − 1), the mean true MIC was 3.07 (SD 0.03), and if the baseline trait level was relatively high (i.e., mean ${\theta }_{\text{T}1}$ = 1), the mean true MIC was 2.63 (SD 0.02). The true MIC varying with the baseline trait level relates to skewness of the PROM scores. The baseline PROM score was positively skewed (to the right) if the trait level was relatively low, whereas the baseline PROM score was negatively skewed (to the left) if the trait level was relatively high. Skewness implies that relatively more simulees sat at or near the extremes of the distribution, where the possibility to improve the PROM score was limited. Therefore, the mean PROM change score if all simulees improved 0.5 $\Delta \theta$ on the latent trait, decreased if the mean baseline score was relatively low or relatively high. Moreover, in the high trait level samples the problem was aggravated by increased skewness of the follow-up score, whereas in the low trait level samples the problem was somewhat alleviated because the follow-up score was less skewed than the baseline score.

### Effect of PSB on the MIC

#### Mean change MIC

**Fig. 1:**
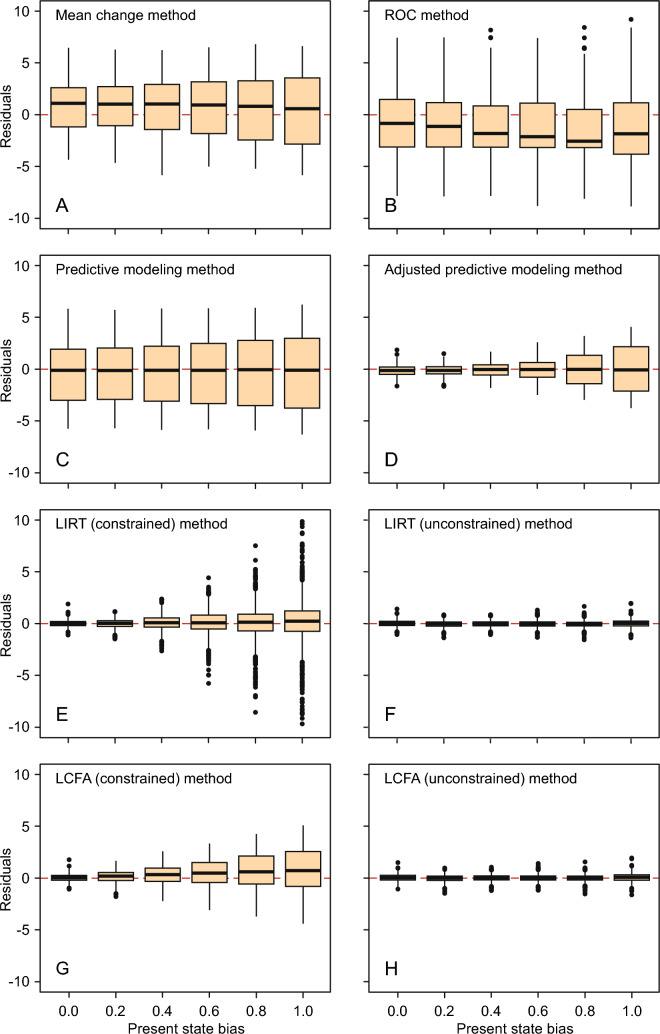
Distributions of MIC residuals (MIC estimate minus true MIC) by MIC estimation method and degree of present state bias. The horizontal dashed lines indicate the true residual value. Note that seventeen outliers are not shown in panel E

Figure 1 provides an overview of the effect of PSB on the MIC estimates. On average, MIC_MC_ (Fig. 1, panel A) slightly overestimated the true MIC across all degrees of PSB. Table 3 confirms that MIC_MC_ was positively biased across all degrees of PSB, the bias being less than one point on a 30-points PROM scale. Given the Monte Carlo standard errors of the bias statistics, the bias was statistically significant [29]. Furthermore, Table 3 shows that the SD of the residuals and the MSE increased with increasing degrees of PSB, suggesting a decrease in the estimates’ precision.

**Table 3:** Performance measures of the MIC estimation by MIC method (Monte Carlo standard errors in parenthesis)

MIC method/statistic	Degree of present state bias					
MIC_MC_	0	0.2	0.4	0.6	0.8	1
N simulations	540	540	540	540	540	540
Mean residuals (bias)	0.92 (0.11)	0.96 (0.11)	0.90 (0.11)	0.79 (0.12)	0.63 (0.13)	0.49 (0.14)
Standard deviation of residuals	2.49 (0.08)	2.49 (0.08)	2.66 (0.08)	2.81 (0.09)	3.03 (0.09)	3.30 (0.10)
Mean squared error	7.02 (0.34)	7.12 (0.35)	7.87 (0.36)	8.51 (0.37)	9.57 (0.41)	11.09 (0.45)
MIC_ROC_						
N simulations	540	540	540	540	540	540
Mean residuals (bias)	− 0.59 (0.13)	− 0.76 (0.13)	− 0.97 (0.13)	− 1.15 (0.14)	− 1.32 (0.14)	− 1.19 (0.15)
Standard deviation of residuals	3.03 (0.09)	2.93 (0.09)	2.98 (0.09)	3.21 (0.10)	3.32 (0.10)	3.50 (0.11)
Mean squared error	9.55 (0.44)	9.16 (0.41)	9.79 (0.44)	11.59 (0.51)	12.73 (0.53)	13.63 (0.65)
MIC_PM_						
N simulations	540	540	540	540	540	540
Mean residuals (bias)	− 0.27 (0.12)	− 0.27 (0.12)	− 0.26 (0.13)	− 0.25 (0.13)	− 0.24 (0.14)	− 0.24 (0.15)
Standard deviation of residuals	2.90 (0.09)	2.89 (0.09)	2.98 (0.09)	3.09 (0.09)	3.24 (0.10)	3.40 (0.10)
Mean squared error	8.45 (0.37)	8.41 (0.36)	8.91 (0.37)	9.58 (0.38)	10.51 (0.41)	11.57 (0.45)
MIC_APM_						
N simulations	540	540	540	540	540	540
Mean residuals (bias)	− 0.13 (0.02)	− 0.12 (0.02)	− 0.07 (0.03)	− 0.06 (0.05)	− 0.04 (0.07)	− 0.05 (0.09)
Standard deviation of residuals	0.51 (0.02)	0.52 (0.02)	0.69 (0.02)	1.08 (0.03)	1.58 (0.05)	2.15 (0.07)
Mean squared error	0.27 (0.02)	0.29 (0.02)	0.48 (0.03)	1.16 (0.06)	2.50 (0.10)	4.64 (0.17)
MIC_LIRT_, constrained						
N simulations	540	540	540	540	540	540
Mean residuals (bias)	0.02 (0.01)	0.02 (0.02)	0.07 (0.04)	0.07 (0.06)	− 0.00 (0.10)	0.03 (0.17)
Standard deviation of residuals	0.33 (0.01)	0.46 (0.01)	0.83 (0.03)	1.38 (0.04)	2.23 (0.07)	3.96 (0.12)
Mean squared error	0.11 (0.01)	0.21 (0.01)	0.69 (0.05)	1.90 (0.15)	4.96 (0.59)	15.63 (2.34)
MIC_LIRT_, unconstrained						
N simulations	540	540	540	540	540	540
Mean residuals (bias)	0.03 (0.01)	− 0.04 (0.01)	− 0.02 (0.01)	− 0.03 (0.02)	− 0.04 (0.01)	0.02 (0.02)
Standard deviation of residuals	0.32 (0.01)	0.32 (0.01)	0.31 (0.01)	0.35 (0.01)	0.34 (0.01)	0.40 (0.01)
Mean squared error	0.10 (0.01)	0.10 (0.01)	0.10 (0.01)	0.12 (0.01)	0.12 (0.01)	0.16 (0.01)
MIC_LCFA_, constrained						
N simulations	540	540	540	540	540	536
Mean residuals (bias)	0.04 (0.02)	0.14 (0.03)	0.29 (0.04)	0.41 (0.06)	0.53 (0.08)	0.62 (0.09)
Standard deviation of residuals	0.35 (0.01)	0.59 (0.02)	0.97 (0.03)	1.43 (0.04)	1.82 (0.06)	2.17 (0.07)
Mean squared error	0.12 (0.01)	0.37 (0.02)	1.03 (0.05)	2.20 (0.11)	3.58 (0.16)	5.08 (0.23)
MIC_LCFA_, unconstrained						
N simulations	540	540	540	540	540	540
Mean residuals (bias)	0.05 (0.01)	− 0.01 (0.01)	0.02 (0.01)	0.01 (0.02)	0.01 (0.02)	0.07 (0.02)
Standard deviation of residuals	0.34 (0.01)	0.34 (0.01)	0.33 (0.01)	0.36 (0.01)	0.35 (0.01)	0.41 (0.01)
Mean squared error	0.12 (0.01)	0.12 (0.01)	0.11 (0.01)	0.13 (0.01)	0.12 (0.01)	0.18 (0.01)

#### ROC-based MIC

Figure 1 (panel B) and Table 3 show that, on average, MIC_ROC_ slightly underestimated the true MIC, and that the estimate was also rather imprecise across all degrees of PSB

#### Predictive modeling MIC

Figure 1 (panel C) and Table 3 show that, on average, MIC_PM_ appeared to have minimal bias due to PSB, but considerable imprecision, which slightly increased with increasing PSB

#### Adjusted predictive modeling MIC

MIC_APM_ appeared to have minimal bias due to PSB (Table 3). The precision of the estimate was relatively high up to PSB = 0.4, but the precision decreased with increasing PSB (Fig. 1, panel D).

#### LIRT-based MIC, constrained

MIC_LIRT_ showed practically no bias due to PSB, irrespective of the degree of PSB (Table 3 and Fig. 1, panel E). However, beyond PSB = 0.2, the MSE increased progressively, suggesting loss of precision of the estimate. Moreover, in the PSB > 0.6 situation, there was a sharp increase in “outlying” estimates, probably related to model misspecification. In four samples no estimates were returned due to non-convergence.

#### LIRT-based MIC, unconstrained

In contrast to the constrained MIC_LIRT_, the unconstrained version appeared to recover the true MIC with practically no bias and high precision across all degrees of PSB (Fig. 1, panel F, and Table 3).

#### LCFA-based MIC, constrained

With increasing PSB, MIC_LCFA_ showed some minor increase in bias but a significant decrease in precision (increase in SD and MSE; Table 3 and Fig. 1, panel G).

#### LCFA-based MIC, unconstrained

Unlike the constrained MIC_LCFA_, and alike the unconstrained MIC_LIRT_, the unconstrained version of MIC_LCFA_ proved to recover the true MIC with practically no bias and high precision across all degrees of PSB (Fig. 1, panel H, and Table 3).

### Effect of PSB relative to other sample characteristics

The results of the regression analyses are summarized in Table 4 (detailed results are provided in the Online Resource, Sect. 2). The unconstrained versions of MIC_LIRT_ and MIC_LCFA_ are not included in Table 4 because none of the sample characteristics (including PSB) affected these estimates. Most often, the MIC estimates were impacted by the proportion improved and $\overline{{\theta }_{\text{T}1}}$. PSB affected the estimation of MIC_APM_ and the constrained versions of MIC_LIRT_ and MIC_LCFA_, mainly through interactions with other sample characteristics, but not the other MIC estimates. The reliability of the TR item did not impact the MIC estimates at all. Given the explained variances, the simulation parameters explained a considerable amount of variance (71%—92%) in the MIC estimates.

**Table 4:** Results of the regression analyses: simulation parameters that explained the variance in the MIC residuals, and explained variance (adjusted R^2^), by MIC method

MIC method	Simulation parameters						Explained variance (R^2^)
	($\overline{{\theta }_{\text{T}1}}$) (1)	SD $\Delta \theta$ (2)	${\theta }_{\text{T}1}$–$\Delta \theta$ correlation (3)	Proportion improved (4)	TR reliability (5)	PSB (6)	
MIC_MC_	I: 1×4			M, I: 1×4			0.91
MIC_ROC_	I: 1×4			M, I: 1×4			0.74
MIC_PM_				M			0.92
MIC_APM_	M, I: 1×3		I: 1×3	I: 4×6		M, I: 4×6	0.82
MIC_LIRT_, constrained		I: 2×3×6, 2×3×4×6	I: 3×6, 2×3×6, 3×4×6, 2×3×4×6	I: 3×4×6, 2×3×4×6		I: 3×6, 2×3×6, 3×4×6, 2×3×4×6	0.71
MIC_LCFA_, constrained	I: 1×6	I: 2×3×6, 2×3×4×6	I: 2×3×6, 3×4×6, 2×3×4×6	I: 4×6, 2×3×6, 3×4×6, 2×3×4×6		M, I: 1×6, 4×6, 2×3×6, 3×4×6, 2×3×4×6	0.83

Simulation parameters: see Table 1, M = main effect, I: interaction effect

## Discussion

### Effect of PSB on the MIC

PSB did not clearly impact MIC_MC_, MIC_ROC_ and MIC_PM_ but the estimates were nevertheless biased by other factors, notably the proportion improved and $\overline{{\theta }_{\text{T}1}}$. MIC_APM_ appeared to become less precise with increasing PSB, an effect that was mediated predominantly by the proportion improved. The constrained versions of MIC_LIRT_ and MIC_LCFA_ also clearly suffered from a decrease in precision with increasing PSB, an effect that was partly caused by model misspecification and partly mediated by the proportion improved and other sample characteristics. The constrained MIC_LCFA_ showed some progressive positive bias, up to 0.62 points, with increasing degrees of PSB, an effect that was real given the Monte Carlo standard errors (Table 3). We have no explanation why this occurred in the constrained MIC_LCFA_ and not in the constrained MIC_LIRT_.

Our most remarkably finding was that the unconstrained versions of MIC_LIRT_ and MIC_LCFA_ appeared not to be affected by PSB at all (nor by any other sample characteristic).

To understand why the unconstrained versions of MIC_LIRT_ and MIC_LCFA_ recovered the true MIC even if PSB was complete, we need to consider the response to the transition item in the general “item factor analysis” framework [30]. We will elaborate the explanation for the LIRT framework [22], but the explanation is analogous for the LCFA framework [23, 31].

The response to the transition item as an indicator of the latent change can mathematically be described as a function $f\left(\text{TR}\right)$—typically a logit function in LIRT—of the latent states at T1 (${\theta }_{\text{T}1}$) and T2 (${\theta }_{\text{T}2}$):

2 $$f\left(\text{TR}\right)={{\upalpha }}_{\text{TR}1}\times{\theta }_{\text{T}1}+{{\upalpha }}_{\text{TR}2}\times{\theta }_{\text{T}2}+{\updelta }_{\text{TR}}$$

where ${{\upalpha }}_{\text{TR}1}$ and ${{\upalpha }}_{\text{TR}2}$ represent regression coefficients (denoted slope parameters in IRT), and ${\updelta }_{\text{TR}}$ represents the intercept [23].

In a previous study we found the following relationship between $q$ (i.e., the degree of PSB) and the regression coefficients ${{\upalpha }}_{\text{TR}1}$ and ${{\upalpha }}_{\text{TR}2}$ (for a mathematical proof see the Online Resource, Sect. 3)^7^:

$$q= \frac{{{\upalpha }}_{\text{TR}1}}{{{\upalpha }}_{\text{TR}2}}+1$$

Because ${{\upalpha }}_{\text{TR}1}={{\upalpha }}_{\text{TR}2}\times(q-1)$, it follows that Eq. (2) can be rewritten as:

3 $$\begin{aligned} f\left( {{\text{TR}}} \right) & = {\upalpha }_{{\text{TR}}2} \times \left( {q - 1} \right) \times \theta_{{\text{T}}1} + {\upalpha }_{{\text{TR}}2} \times \theta_{{\text{T}}2} + {\updelta }_{{\text{TR}}} \quad {\text{and:}} \\ f\left( {{\text{TR}}} \right) & = {\upalpha }_{{\text{TR}}2} \times \left( {\theta_{{\text{T}}2} - \left( {1 - q} \right) \times \theta_{{\text{T}}1} } \right) + {\updelta }_{{\text{TR}}} \\ \end{aligned}$$

The expression ${\theta }_{\text{T}2}-\left(1-q\right)\times{\theta }_{\text{T}1}$, within Eq. (3), represents an alternative expression of the weighted change in Eq. (1) (for proof see the Online Resource, Sect. 4). If $q=0$ the weighted change equals the true change (i.e., ${\theta }_{\text{T}2}-{\theta }_{\text{T}1}$); if $q=1$ the weighted change equals the present state (i.e., ${\theta }_{\text{T}2}$). Equation (3) clearly indicates that the transition item is an indicator of the weighted change with slope parameter ${{\upalpha }}_{\text{TR}2}$ and intercept ${\updelta }_{\text{TR}}$, independent of the degree of PSB. This is why the MIC threshold on the latent variable underlying the transition item is correctly estimated using ${{\upalpha }}_{\text{TR}2}$ and ${\updelta }_{\text{TR}}$, irrespective of the degree of PSB (provided that ${{\upalpha }}_{\text{TR}1}$ and ${{\upalpha }}_{\text{TR}2}$ are not constrained).^8^

Importantly, if the degree of PSB is large, patients’ responses to the transition question may not reflect their true change, and the reported proportion improved may not agree with the true proportion improved. Nevertheless, using these (faulty) transition ratings, we are able to recover the correct MIC as if there had been no PSB.

### Limitations

Simulation studies always require assumptions about how things play out in the real world. The simulation of something elusive as present state bias was quite a challenge. In particular, we made two key assumptions. First, we assumed that patients’ collective memory of their baseline state (the reference point in case of PSB) is unbiased, in the sense that patients do not collectively cherish a too favorable (or too unfavorable) memory of their baseline state. Second, we assumed that patients use the same personal thresholds to evaluate their perceived change, irrespective of the degree of PSB. If any of these assumptions are violated, the estimated MIC will be biased.^9^ Unfortunately, we just have to accept the two key assumptions mentioned above, because there is currently no way to test whether or not the assumptions are met in item response data. Moreover, without adopting these assumptions, it will be impossible to estimate MIC values using any of the methods examined. Perhaps, future qualitative research (cognitive interviewing) might shed more light on this matter.

Furthermore, we did not include response shift in the simulations and analyses, as we used invariant item parameters across the time points. The effect of response shift on MIC estimation needs further simulation research.

## Conclusions and recommendations

PSB does not impact MICs estimated using the mean change method, the ROC method and the predictive modeling method. Yet, these methods can no longer be recommended because of bias due to the proportion improved (and baseline severity). The MIC based on adjusted predictive modeling can safely be used up until 40% PSB (i.e., PSB < 0.4). The MICs based on LIRT and LCFA provide unbiased and precise estimates, irrespective of the degree of PSB—but only if the transition item slope parameters (in LIRT) or factor loadings (in LCFA) are freely estimated. As the unconstrained MICs also perfectly recover the MIC if there is no PSB, we recommend not to apply these constraints anymore (see the Online Resource, Sect. 6, for the R-code for the recommended methods: adjusted predictive modeling (APM), unconstrained LIRT, and unconstrained LCFA).

## Supplementary Information

Below is the link to the electronic supplementary material.

Supplementary file 1 (DOCX 381 kb)

## Data Availability

The R-code used to simulate and analyze samples is provided in the Supplementary material.

## Footnotes

1 Relevant change can occur in two directions, improvement or deterioration. For the sake of simplicity, we limit our account to improvement.

2 The choice for 10 items and 4 response options is arbitrary but inconsequential as to the principles of the simulation and analysis methods.

3 Note that higher trait levels represent “better” states, and positive latent change scores represent improvement.

4 The choice for 0.3 and 0.5 was based on a study of 4 empirical datasets, in which the reliability of the transition ratings appeared to vary between 0.27 and 0.48 [12].

5 In a fitted LIRT or LCFA model, $\overline{{\theta }_{\text{T}1}}$ is typically set to 0, in which case ${\theta }_{\text{T}2}-\overline{{\theta }_{\text{T}1}}={\theta }_{\text{T}2}$, and the value zero (which equals $\overline{{\theta }_{\text{T}1}}$) becomes the natural baseline reference point. However, during the simulation procedure, we also used different values of $\overline{{\theta }_{\text{T}1}}$ (i.e., − 1 and 1, because the item parameters were fixed) and we needed to take this into account when simulating ${\Delta \theta }_{w}$.

6 Simulating the SD of the *q-*variable to be (0.5 − |(0.5 − *q*)|)/4 ensures maximum variability of normally distributed individual *q*-values while most values fall within the 0–1 range.

7 We found that PSB could be quantified by $\frac{{\uplambda }_{\text{TR}1}}{{\uplambda }_{\text{TR}2}}+1$ in an LCFA framework [9]. Under the specific specifications of the LCFA-model used for estimating the MIC, $\frac{{\uplambda }_{\text{TR}1}}{{\uplambda }_{\text{TR}2}}$ is identical to $\frac{{{\upalpha }}_{\text{TR}1}}{{{\upalpha }}_{\text{TR}2}}$ in the LIRT-model [23].

8 In the LIRT framework the MIC is estimated as $-\frac{{\updelta }_{\text{TR}}}{{{\upalpha }}_{\text{TR}2}}$, whereas in the LCFA framework the MIC is estimated as $\frac{{\uptau }_{\text{TR}}}{{\uplambda }_{\text{TR}2}}$, where ${\uptau }_{\text{TR}}$ represents the threshold of the transition item, and ${\uplambda }_{\text{TR}2}$ represents the factor loading of the transition item on the T2 factor.

9 Additional simulations revealed that, in the presence of PSB, patients’ systematic overestimation of their baseline state results in overestimation of the MIC whereas underestimation of the baseline state results in underestimation of the MIC. If patients would apply higher personal thresholds to evaluate their change in the presence of PSB than they would in the absence of it, the MIC will be overestimated. If patients would apply lower thresholds, the MIC will be underestimated.

## Code availability

The R-code is provided in the Supplementary material.
