# Supplementary material for: Effect of present state bias on minimal important change estimates: a simulation study
Source: Qual Life Res. 2024 Aug 22;33(11):2963–73. doi: 10.1007/s11136-024-03763-4 (PMC11541299; doi:10.1007/s11136-024-03763-4)
Supplement: Supplementary file 1 — (DOCX 381 kb) [file 11136_2024_3763_MOESM1_ESM.docx]

**Online Supplement**

**Effect of present state bias on minimal important change estimates; a simulation study**

Berend Terluin, Piper Fromy, Andrew Trigg, Caroline B Terwee, Jakob B Bjorner

Quality of Life Research, 2024

1. Item parameters for simulation 2

2. Results regression analyses 3

3. Proof of present state bias $q= \frac{\alpha_{\mathrm{TR}1}}{\alpha_{TR2}}+1$ 11

4. Proof of two equivalent expressions of weighted change 13

5. R-code

R-code for the simulations 14

R-code for the analyses 28

6. R-code for the recommended methods 37

Adjusted predictive modeling 37

LIRT method, unconstrained 41

LCFA method, unconstrained 42

**1. Item parameters for simulation**^[[1]](#footnote-1)^

Item response theory (IRT) provides a probabilistic model for understanding persons’ responses to the items of a PROM as an interaction between person characteristics and item characteristics. The person characteristic of interest is their standing on the construct the PROM is purported to measure (e.g., the person’s level of physical functioning). Relevant item characteristics are ‘slope’ and ‘location’. In the graded response model, the slope parameter (α) reflects the strength of an item as an indicator of the construct. The location parameter (β) is a characteristic of the item’s response options, and reflects the level of the construct at which the probability to endorse a particular response option (or a higher option) equals 50%. For items with 4 response options there are 3 location parameters (β1, β2, β3).

The item parameters were simulated as follows: The β2 parameters consisted of a series of numbers between -0.8 and +0.8 with intervals of 0.4. The β1 parameters were based on β2 minus 1 plus a random distribution of β2/4. The β3 parameters were based on β2 plus 1 plus a different random distribution of β2/4. The α parameters consisted of 1.7 + β2/2, randomly distributed.

Item parameters of hypothetical questionnaire with 10 items, reliability ~0.86

| **Item** | **α** | **β1** | **β2** | **β3** |
| --- | --- | --- | --- | --- |
| 1 | 2.1 | -1.9 | -0.8 | 0.4 |
| 2 | 1.5 | -1.7 | -0.8 | 0.0 |
| 3 | 1.3 | -1.6 | -0.4 | 0.7 |
| 4 | 1.9 | -1.4 | -0.4 | 0.7 |
| 5 | 1.7 | -0.8 | 0.0 | 1.0 |
| 6 | 1.9 | -0.8 | 0.0 | 1.2 |
| 7 | 2.1 | -0.5 | 0.4 | 1.3 |
| 8 | 1.5 | -0.6 | 0.4 | 1.2 |
| 9 | 1.7 | -0.3 | 0.8 | 1.7 |
| 10 | 1.3 | -0.4 | 0.8 | 1.8 |
| **Mean** | **1.7** | **-1.0** | **0.0** | **1.0** |

**2. Results regression analyses**

***Mean change MIC****.* Multivariate linear regression analysis showed that 98% (adjusted R^2^) of the variance of the MIC_mean_ residuals was explained by the simulation parameters and their interactions. After backward elimination of little contributing interactions and determinants, a model with proportion improved and the interaction between proportion improved and $\bar{\theta_{T1}}$ accounted for 91% of the variance (Table S1).

**Table S1**. Final regression model explaining MIC_mean_ residuals

| **Coefficients** | **Estimate** | **SE** | **t value** | **P** |
| --- | --- | --- | --- | --- |
| Intercept | -4.608 | 0.034 | -136.00 | 0.0000 |
| Prop. improved | 10.780 | 0.061 | 177.14 | 0.0000 |
| Prop. improved*$\bar{\theta_{T1}}$ | -1.048 | 0.033 | -31.96 | 0.0000 |

SE = standard error, $\bar{\theta_{T1}}$ = mean latent trait at T1

Fig. S1 shows that, if the proportion improved was 0.5, MIC_mean_ showed some (0.86 points) overestimation of the true MIC. However, the overestimation increased with proportions improved > 0.5, whereas the overestimation eventually turned into underestimation with proportions improved much less than 0.5. $\bar{\theta_{T1}}$ appeared to have some dampening effect on the bias caused by the proportion improved, especially if proportion improved was > 0.5.


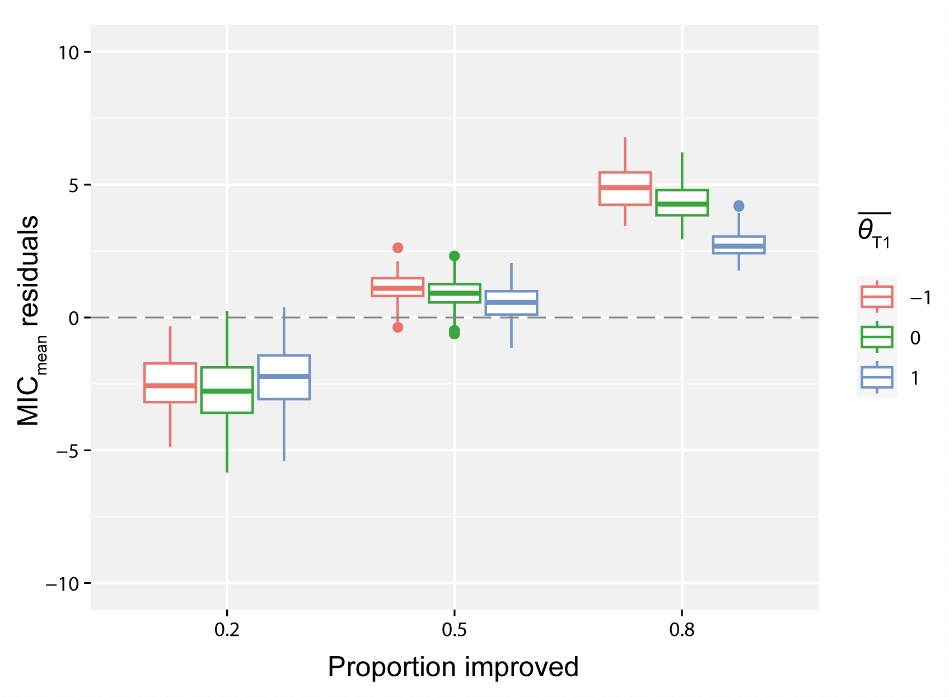


**Fig. S1**. Distributions of MIC_mean_ residuals by proportion improved and mean latent trait at T1 ($\bar{\theta_{T1}}$). The dashed line indicates the “true” value of the residuals

***ROC-based MIC****.* The simulation parameters and their interactions explained 82% of the variance of the MIC_ROC_ residuals. After backward elimination, proportion improved and the interaction between proportion improved and $\bar{\theta_{T1}}$ explained 74% of the variance (Table S2). Fig. S2 shows that MIC_ROC_, on average, slightly underestimated the true MIC if the proportion improved was 0.5, but if the proportion improved was smaller, the underestimation worsened, whereas if the proportion improved was much greater than 0.5, MIC_ROC_ tended to overestimate the true MIC, except in the presence of relatively low baseline severity.

**Table S2**. Final regression model explaining MIC_ROC_ residuals

| **Coefficients** | **Estimate** | **SE** | **t value** | **P** |
| --- | --- | --- | --- | --- |
| Intercept | -5.679 | 0.065 | -87.95 | 0.0000 |
| Prop. improved | 9.367 | 0.116 | 80.78 | 0.0000 |
| Prop. improved*$\bar{\theta_{T1}}$ | -3.269 | 0.062 | -52.32 | 0.0000 |

SE = standard error, $\bar{\theta_{T1}}$ = mean latent trait at T1


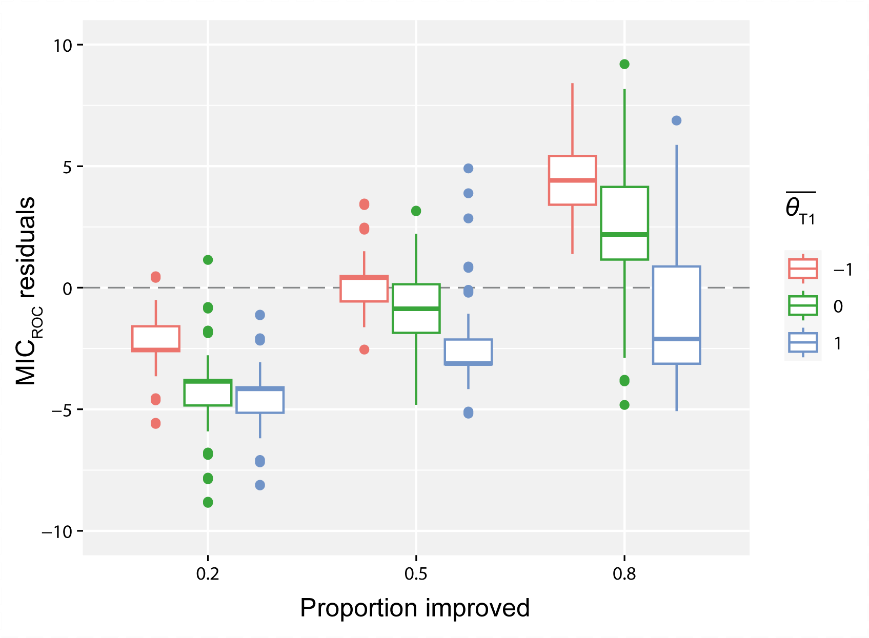


**Fig. S2**. Distributions of MIC_ROC_ residuals by proportion improved and mean latent trait at T1 ($\bar{\theta_{T1}}$). The dashed line indicates the “true” value of the residuals

***Predictive modeling MIC****.* Regression analysis showed that 99% of the variance in the MIC_PM_ residuals was explained by all simulation parameters and their interactions. After backward elimination, 92% of the variance was explained by the proportion improved (Table S3). $\bar{\theta_{T1}}$ just failed to meet the requirement of explaining more than 2% of the residuals’ variance. Fig. S3 shows how MIC_PM_ is biased by the proportion improved.

**Table S3**. Final regression model explaining MIC_PM_ residuals

| **Coefficients** | **Estimate** | **SE** | **t value** | **P** |
| --- | --- | --- | --- | --- |
| Intercept | -6.292 | 0.035 | -180.9 | 0.0000 |
| Prop. improved | 12.075 | 0.062 | 193.2 | 0.0000 |

SE = standard error


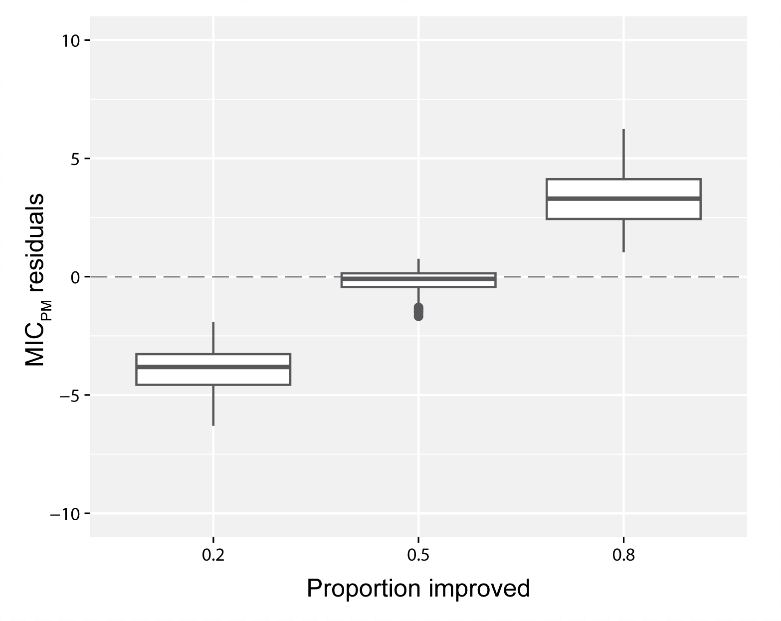


**Fig. S3**. Distributions of MIC_PM_ residuals by proportion improved. The dashed line indicates the “true” value of the residuals

***Adjusted predictive modeling MIC****.* The variance of the MIC_APM_ residuals was for 89% explained by the simulation parameters and their interactions. After backward elimination, we ended up with a model with 6 determinant terms, explaining 84% of the variance. A plot of the MIC residuals against the two most influential simulation parameters, PSB and proportion improved (Fig. S4), suggested a non-linear effect of PSB. Therefore, we added PSB-squared to the regression model, which increased the explained variance to 86%. After a new round of elimination of less contributing determinants, a model remained with 4 determinant terms, of which 2 two-way interactions, explaining 82% of the variance (Table S4). From PSB = 0.4 upward, PSB caused biased MIC_APM_ estimates, the direction of the bias depending on the proportion improved (Fig. S4).

**Table S4**. Final regression model explaining MIC_APM_ residuals

| **Coefficients** | **Estimate** | **SE** | **t value** | **P** |
| --- | --- | --- | --- | --- |
| Intercept | -0.105 | 0.013 | -7.96 | 0.0000 |
| $\bar{\theta_{T1}}$ | -0.421 | 0.016 | -26.39 | 0.0000 |
| PSB^2^ | -4.316 | 0.045 | -95.90 | 0.0000 |
| PSB^2^ * Prop. improved | 8.785 | 0.074 | 119.33 | 0.0000 |
| $\bar{\theta_{T1}}$ * $\theta_{T1}$-$\Delta\theta$ correlation | -1.147 | 0.045 | -25.41 | 0.0000 |

SE = standard error, $\bar{\theta_{T1}}$ = mean latent trait at T1, PSB^2^ = present state bias squared, $\theta_{T1}$-$\Delta\theta$ correlation = correlation between latent trait T1 and latent change


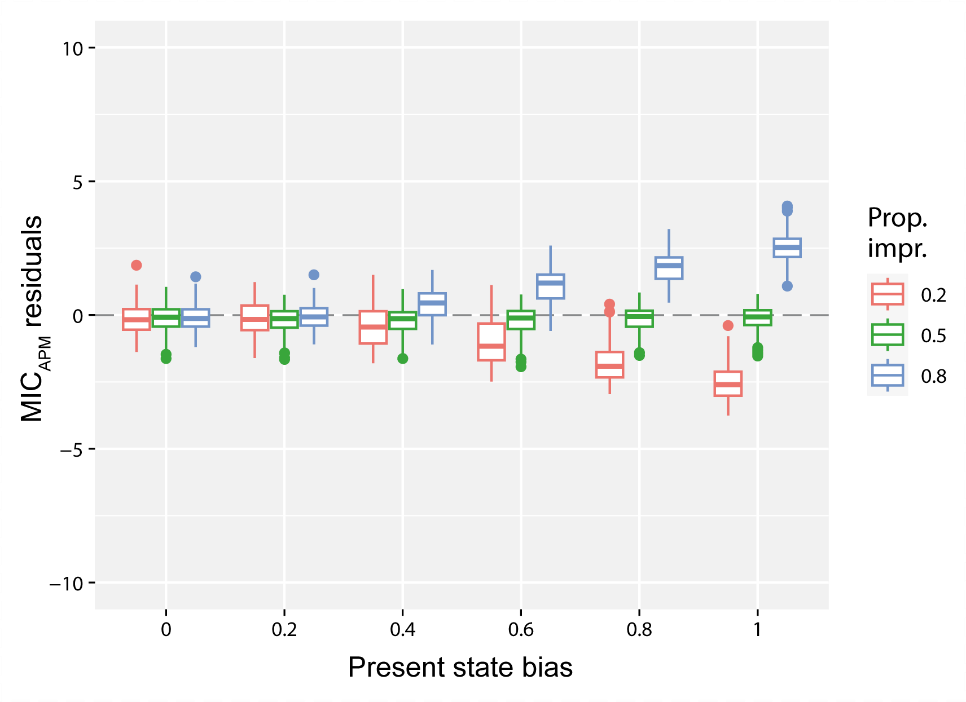


**Fig. S4**. Distributions of MIC_APM_ residuals by present state bias and proportion improved. The dashed line indicates the “true” value of the residuals

***LIRT-based MIC****.* All simulation parameters and all their interactions explained 74% (adjusted R^2^) of the variance of the residuals of the *constrained* MIC_LIRT_ estimates. Backward elimination resulted in a rather complex model with five determinant terms, explaining 71% of the residuals’ variance. A plot of the residuals against PSB and the most influential determinants suggested a non-linear effect of PSB. Therefore, we added PSB-squared to the model. After further backward elimination, a model with four determinant terms remained, including one 2-way interaction term, two 3-way interaction terms, and one 4-way interaction term (Table S5). The model explained 71% of the residuals’ variance. The most influential simulation parameters were the correlation between $\theta_{T1}$ and $\Delta\theta$ and proportion improved, in interaction with PSB-squared (Fig. S5). The correlation between $\theta_{T1}$ and $\Delta\theta$ determined the spread of the estimates (Fig. S5, upper panel), whereas the proportion improved determined the direction of the bias (Fig. S5, lower panel). As long as the correlation was zero or the proportion improved was 0.5, the MIC estimates showed little bias and imprecision. Proportions improved < 0.5 caused overestimation and proportions improved > 0.5 caused underestimation of the MIC if PSB was > 0.2.

With respect to the MICs based on LIRT-models with *unconstrained* TR slope parameters, the simulation parameters and all their interactions explained none of its residuals’ variance (R^2^ = 0.00).

**Table S5**. Final regression model explaining MIC_LIRT_ residuals (constrained method)

| **Coefficients** | **Estimate** | **SE** | **t value** | **P** |
| --- | --- | --- | --- | --- |
| Intercept | 0.082 | 0.022 | 3.78 | 0.0002 |
| $\theta_{T1}$-$\Delta\theta$ cor * PBS^2^ | -42.697 | 1.170 | -36.48 | 0.0000 |
| $\theta_{T1}$-$\Delta\theta$ cor * PBS^2^ * SD $\Delta\theta$ | 25.162 | 1.145 | 21.97 | 0.0000 |
| $\theta_{T1}$-$\Delta\theta$ cor * PBS^2^ * Prop.impr | 90.015 | 2.099 | 42.88 | 0.0000 |
| $\theta_{T1}$-$\Delta\theta$ cor * PBS^2^ * Prop.impr * SD $\Delta\theta$ | -53.928 | 2.057 | -26.22 | 0.0000 |

SE = standard error, Prop.impr = proportion improved, $\theta_{T1}$-$\Delta\theta$ cor = correlation between latent trait T1 and latent change, PSB^2^ = present state bias squared, SD $\Delta\theta$ = standard deviation of the latent change


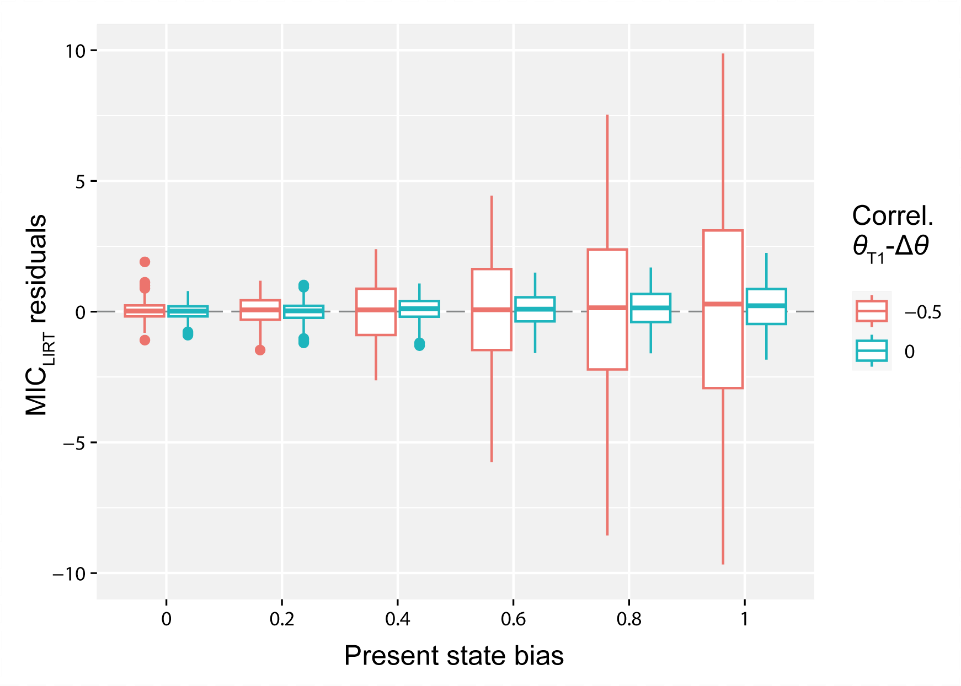


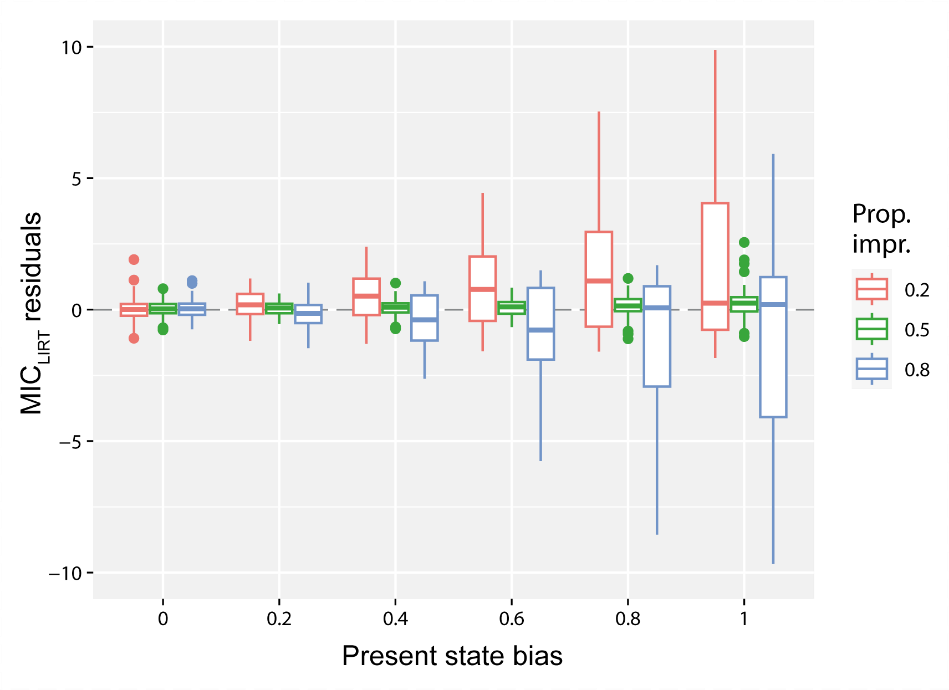


**Fig. S5**. Distributions of MIC_LIRT_ residuals (constrained method) by present state bias and the correlation between $\theta_{T1}$ and $\Delta\theta$ (upper panel) and proportion improved (lower panel). The dashed lines indicate the “true” value of the residuals

***LCFA-based MIC***. The simulation parameters and all their interactions explained 90% (adjusted R^2^) of the variance of the MIC residuals of the *constrained* MIC_LCFA_ estimates. After backwards elimination, 83% of the MIC residuals’ variance was explained by six determinant terms including two 2-way interaction terms, two 3-way interaction terms, and one 4-way interaction term (Table S6). Addition of PSB-squared did not improve the model. The most influential simulation parameters, apart from PSB, were the correlation between $\theta_{T1}$ and $\Delta\theta$ and the proportion improved. The effects of the correlation between $\theta_{T1}$ and $\Delta\theta$ and the proportion improved are shown in Fig. S6. Remarkably, the effect of the correlation between $\theta_{T1}$ and $\Delta\theta$ on the MIC_LCFA_ residuals appeared to be quite different from the effect on the MIC_LIRT_ residuals (compare Fig. S5 and S6, upper panels). Also, the effect of the proportion improved on the MIC_LCFA_ residuals appeared to be even opposite to the effect on the MIC_LIRT_ residuals (compare Fig. S5 and S6, lower panels). This is remarkable because the LIRT- and LCFA-models are thought to be quite similar. Yet, model misspecification (due to negatively constraining parameters/loadings, that are not negatively equal) appears to exert quite different effects.

Regarding the MICs based on LCFA-models with *unconstrained* TR factor loadings, the simulation parameters and all their interactions explained none of its residuals’ variance (R^2^ = 0.00).

**Table S6**. Final regression model explaining MIC_LCFA_ residuals (constrained method)

| **Coefficients** | **Estimate** | **SE** | **t value** | **P** |
| --- | --- | --- | --- | --- |
| Intercept | 0.040 | 0.018 | 2.24 | 0.025 |
| PBS | -4.679 | 0.057 | -81.51 | 0.0000 |
| PSB * $\bar{\theta_{T1}}$ | 0.875 | 0.020 | 43.15 | 0.0000 |
| PBS * Prop.impr | 10.034 | 0.094 | 106.90 | 0.0000 |
| $\theta_{T1}$-$\Delta\theta$ cor * PBS * SD $\Delta\theta$ | -6.814 | 0.144 | -47.22 | 0.0000 |
| $\theta_{T1}$-$\Delta\theta$ cor * PBS * Prop.impr | -8.931 | 0.431 | -20.74 | 0.0000 |
| $\theta_{T1}$-$\Delta\theta$ cor * PBS * Prop.impr * SD $\Delta\theta$ | 20.487 | 0.474 | 43.21 | 0.0000 |

SE = standard error, PSB = present state bias, $\bar{\theta_{T1}}$ = mean latent change at T1, Prop.impr = proportion improved, $\theta_{T1}$-$\Delta\theta$ cor = correlation between latent trait T1 and latent change, SD $\Delta\theta$ = standard deviation of the latent change


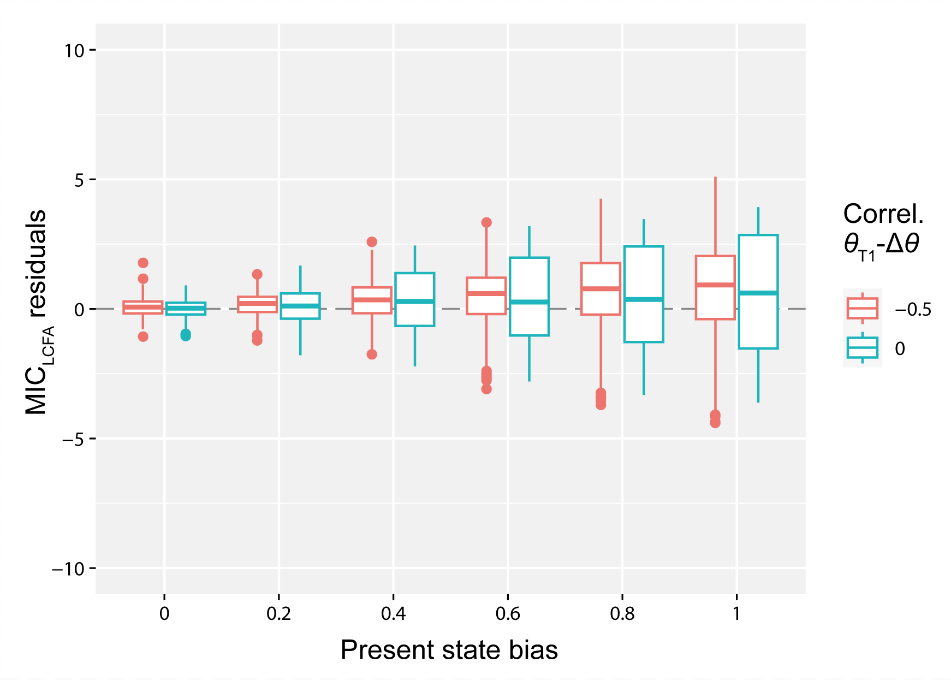


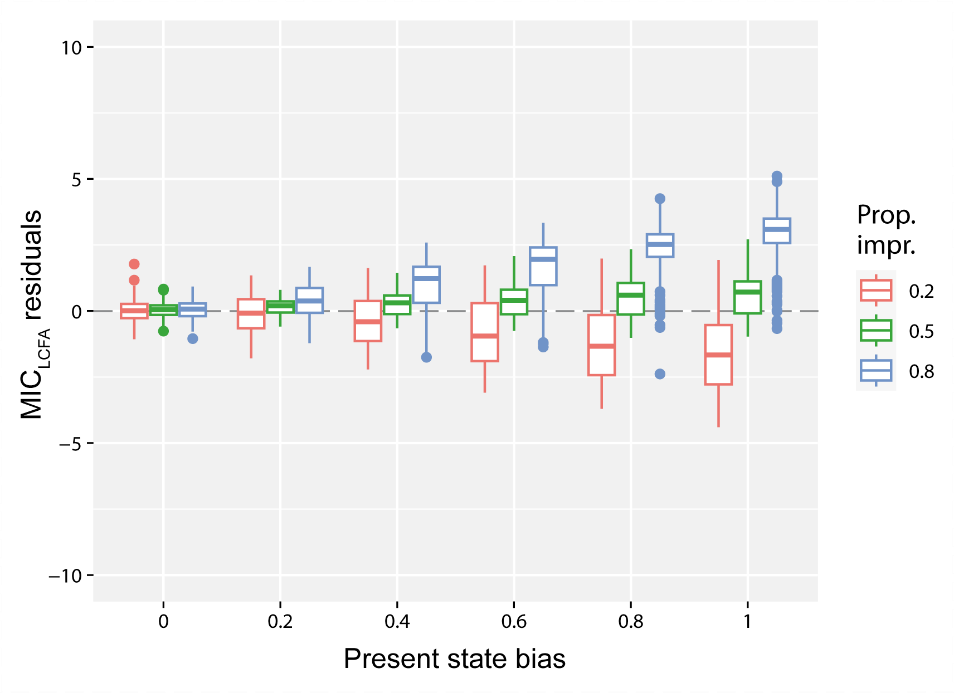


**Fig. S6**. Distributions of MIC_LCFA_ residuals (constrained method) by present state bias and the correlation between $\theta_{T1}$ and $\Delta\theta$ (upper panel) and proportion improved (lower panel). The dashed lines indicate the “true” value of the residuals

**3. Proof of present state bias** $\boldsymbol{q=}\frac{\boldsymbol{\alpha}_{\mathbf{TR}\boldsymbol{1}}}{\boldsymbol{\alpha}_{\mathbf{TR2}}}\boldsymbol{+1}$

In our previous work we empirically derived a formula for a CFA-based PSB-statistic (doi: 10.1016/j.jclinepi.2021.12.024). Below we will derive the same formula mathematically.

In the item factor-analytical framework, the response to a transition item is a function (e.g., a logit function) of two latent variables, $\theta_{T1}$ and $\theta_{T2}$.

$f\left( \mathrm{TR} \right)= \alpha_{TR1}*\theta_{T1}+\alpha_{TR2}*\theta_{T2}+\delta_{\mathrm{TR}}$

$\alpha_{TR1}$ and $\alpha_{TR2}$ represent the regression coefficients of the transition ratings ($\mathrm{TR}$) item on the latent trait factors $\theta_{T1}$ and $\theta_{T2}$, respectively.

Elaboration:

$f\left( \mathrm{TR} \right)= \alpha_{TR2}*\theta_{T2}+\alpha_{TR1}*\theta_{T1}+\delta_{\mathrm{TR}}$

$f\left( \mathrm{TR} \right)= \alpha_{TR2}*(\theta_{T2}+\frac{\alpha_{\mathrm{TR}1}}{\alpha_{TR2}}*\theta_{T1})+\delta_{\mathrm{TR}}$

$f\left( \mathrm{TR} \right)= \alpha_{TR2}*(\theta_{T2}+\frac{\alpha_{\mathrm{TR}1}}{\alpha_{TR2}}*\theta_{T1}+\frac{\alpha_{\mathrm{TR}1}}{\alpha_{TR2}}*\theta_{T2}-\frac{\alpha_{\mathrm{TR}1}}{\alpha_{TR2}}*\theta_{T2})+\delta_{\mathrm{TR}}$

$f\left( \mathrm{TR} \right)= \alpha_{TR2}*(\theta_{T2}+\frac{\alpha_{\mathrm{TR}1}}{\alpha_{TR2}}*\theta_{T2}-\frac{\alpha_{\mathrm{TR}1}}{\alpha_{TR2}}*\theta_{T2}+\frac{\alpha_{\mathrm{TR}1}}{\alpha_{TR2}}*\theta_{T1})+\delta_{\mathrm{TR}}$

$f\left( \mathrm{TR} \right)= \alpha_{TR2}*((\theta_{T2}+\frac{\alpha_{\mathrm{TR}1}}{\alpha_{TR2}}*\theta_{T2})+(-\frac{\alpha_{\mathrm{TR}1}}{\alpha_{TR2}}*\theta_{T2}+\frac{\alpha_{\mathrm{TR}1}}{\alpha_{TR2}}*\theta_{T1}))+\delta_{\mathrm{TR}}$

$f\left( \mathrm{TR} \right)= \alpha_{TR2}*((1+\frac{\alpha_{\mathrm{TR}1}}{\alpha_{TR2}})*\theta_{T2}-\frac{\alpha_{\mathrm{TR}1}}{\alpha_{TR2}}*(\theta_{T2}-\theta_{T1}))+\delta_{\mathrm{TR}}$

as $\Delta\theta=\theta_{T2}-\theta_{T1}$,

$f\left( \mathrm{TR} \right)= \alpha_{TR2}*((1+\frac{\alpha_{\mathrm{TR}1}}{\alpha_{TR2}})*\theta_{T2}-\frac{\alpha_{\mathrm{TR}1}}{\alpha_{TR2}}*\Delta\theta)+\delta_{\mathrm{TR}}$

$f\left( \mathrm{TR} \right)= (1+\frac{\alpha_{\mathrm{TR}1}}{\alpha_{TR2}})*\alpha_{TR2}*\theta_{T2}-\frac{\alpha_{\mathrm{TR}1}}{\alpha_{TR2}}*\alpha_{TR2}*\Delta\theta+\delta_{\mathrm{TR}}$ (1)

Eq. (1) indicates that the response to the transition item is determined by two variables, $\theta_{T2}$ (i.e., the present state) and $\Delta\theta$ (i.e., the true change), which are weighted by $(1+\frac{\alpha_{\mathrm{TR}1}}{\alpha_{TR2}})*\alpha_{TR2}$ and $-\frac{\alpha_{\mathrm{TR}1}}{\alpha_{TR2}}*\alpha_{TR2}$ respectively. Present state bias (PSB) is said to be present if the response to the transition item is more heavily determined by the present state ($\theta_{T2}$) than by the previous state ($\theta_{T1}$) or, in other words, if the response is to some extent determined by the present state ($\theta_{T2}$) instead of the true change ($\Delta\theta$). The degree of PSB (which can vary between 0 and 1) is in fact determined by $\alpha_{TR1}$. In the absence of PSB (i.e., PSB = 0), $\alpha_{TR1}$ equals $-\alpha_{TR2}$, in which case $(1+\frac{\alpha_{\mathrm{TR}1}}{\alpha_{TR2}})*\alpha_{TR2}$ equals zero, and the response to the transition item is solely determined by the true change ($\Delta\theta$) (with regression coefficient $\alpha_{TR2}$). On the other hand, in the presence of complete PSB (i.e., PSB = 1), $\alpha_{TR1}$ equals zero, in which case $-\frac{\alpha_{\mathrm{TR}1}}{\alpha_{TR2}}*\alpha_{TR2}$ equals zero, and the response to the transition item is solely determined by the present state ($\theta_{T2}$) (with regression coefficient $\alpha_{TR2}$).

Eq. (1) shows that two variables, $\theta_{T2}$ and $\Delta\theta$, contribute to the response probability of the TR item. PSB can be described as the relative contribution of $\theta_{T2}$ (the present state) to the response probability. The contribution of $\theta_{T2}$ is weighted by $(1+\frac{\alpha_{\mathrm{TR}1}}{\alpha_{TR2}})*\alpha_{TR2}$, whereas the contribution of $\theta_{T2}$ and $\Delta\theta$ together is weighted by $(1+\frac{\alpha_{\mathrm{TR}1}}{\alpha_{TR2}})*\alpha_{TR2}-\frac{\alpha_{\mathrm{TR}1}}{\alpha_{TR2}}*\alpha_{TR2}$.

PSB, that is the relative contribution of $\theta_{T2}$, can therefore be expressed as:

$PSB= \frac{Weight of \theta_{T2}}{Weight of \theta_{T2} and \Delta\theta}$ or:

$PSB= \frac{(1+\frac{\alpha_{\mathrm{TR}1}}{\alpha_{TR2}})*\alpha_{TR2}}{\left( 1+\frac{\alpha_{\mathrm{TR}1}}{\alpha_{TR2}} \right)*\alpha_{TR2} - \frac{\alpha_{\mathrm{TR}1}}{\alpha_{TR2}}*\alpha_{TR2}}$

Elaborating this Equation, results in:

$PSB= \frac{1+\frac{\alpha_{\mathrm{TR}1}}{\alpha_{TR2}}}{1+\frac{\alpha_{\mathrm{TR}1}}{\alpha_{TR2}} - \frac{\alpha_{\mathrm{TR}1}}{\alpha_{TR2}}}$

$PSB= \frac{1+\frac{\alpha_{\mathrm{TR}1}}{\alpha_{TR2}}}{1}$

$PSB= \frac{\alpha_{\mathrm{TR}1}}{\alpha_{TR2}}+1$

We have now mathematically derived the empirically discovered formula for PSB.

**4. Proof of two equivalent expressions of weighted change**

In the Methods section, we simulated the weighted change (${\Delta\theta}_{w}$), using the following Equation:

${\Delta\theta}_{w}=q*(\theta_{T2}-\bar{\theta_{T1}})+\left( 1-q \right)*\Delta\theta$ (2)

where $q$ represents a variable of weights between 0 and 1, indicating the degree of PSB, and where $\bar{\theta_{T1}}$ represents the mean of the baseline state $\theta_{T1}$.

If $\bar{\theta_{T1}}=0$, which is by default the case in fitted LIRT and LCFA models, Eq. (2) becomes:

${\Delta\theta}_{w}=q*\theta_{T2}+\left( 1-q \right)*\Delta\theta$

as $\Delta\theta=\theta_{T2}-\theta_{T1}$, we get:

${\Delta\theta}_{w}=q*\theta_{T2}+\left( 1-q \right)*(\theta_{T2}-\theta_{T1})$

${\Delta\theta}_{w}=q*\theta_{T2}+\left( 1-q \right)*\theta_{T2}-\left( 1-q \right)*\theta_{T1}$

as $q*\theta_{T2}+\left( 1-q \right)*\theta_{T2}=\theta_{T2}$, we get:

${\Delta\theta}_{w}=\theta_{T2}-(1-q)*\theta_{T1}$

Here is the alternative expression of the weighted change, featuring in Eq. (3) in the main text.

**5. R-code**

#############################################################################

#### ####

#### SIMULATION/ESTIMATION OF MICs WITH PSB ####

#### ####

#############################################################################

# This code simulates 2 datasets (baseline, T1; follow-up, T2) for a sample

# of N persons, using the same hypothetical questionnaire (i.e., the same

# items with the same item parameters).

# The transition ratings are simulated as follows: A set of meaningful change

# (individual MICs) thresholds are simulated as normal variables with a mean

# of 0.5. This implies that THE true MIC is 0.5 theta change.

# Note that this threshold determines the proportion of improved patients in

# conjunction with the mean change.

# We assume that patients rate their transition rating based on a comparison

# between their perceived change and their individual MIC.

# We simulated the patients' perceived change scores based on the weighted

# change between T1 and T2, supplemented with measurement error.

# The weighted change indicates present state bias.

# Acquire packages

library(mirt) # use version 1.40 or higher

library(lavaan)

library(semTools)

library(e1071)

library(ggplot2)

library(psych)

library(polycor)

library(pROC)

library(MASS)

library(beepr)

rm(list=ls(all=TRUE)) # remove all objects

### SIMULATE A SET OF ITEM PARAMETERS

# The b2 parameters (the 'middle' location parameters) consist of a series

# of numbers between -0.8 and +0.8 with intervals of 0.4. The b1 parameters

# (the first location parameters) are based on b2 minus 1 plus a random

# distribution of b2/4. The b3 parameters (the third location parameters)

# are based on b2 plus 1 plus a different random distribution of b2/4.

# The a parameters consist of 1.7 + b2/2, randomly distributed.

set.seed(12345)

b2 <- c(-0.8, -0.8, -0.4, -0.4, 0, 0, 0.4, 0.4, 0.8, 0.8)

bc <- b2/4

b1 <- b2 - 1 + sample(bc)

b3 <- b2 + 1 + sample(bc)

a1 <- sample( 1.7+b2/2 )

cf.simb <- as.matrix( data.frame(a1,b1,b2,b3) )

round(cf.simb, 3)

round(colMeans(cf.simb), 3)

cf.simb <- as.data.frame(cf.simb)

# Transform b-parameters to d-parameters ('mirt' works with d-parameters)

# difficulty (b) = easiness (d) / -a

cf.sim <- cf.simb

colnames(cf.sim) <- c("a1","d1","d2","d3")

cf.sim$d1 <- -cf.simb$b1*cf.sim$a1

cf.sim$d2 <- -cf.simb$b2*cf.sim$a1

cf.sim$d3 <- -cf.simb$b3*cf.sim$a1

# round(cf.sim, 3)

# Simulate dataset using 'mirt'

a1 <- as.matrix(cf.sim[ , 1])

d1 <- as.matrix(cf.sim[ , -1])

# Create a dataset and check reliability

# Create dataset with theta = N(0,1)

set.seed(12345)

theta.sim <- as.matrix( rnorm(20000, 0, 1) )

dat <- simdata(a1, d1, 20000, itemtype="graded", Theta=theta.sim)

dat <- as.data.frame(dat)

round( psych::alpha(dat)$total$raw_alpha, 3 ) # Cronbach's alpha

#############################################################################

## SET THE PARAMETERS FOR THE SIMULATIONS

nr=5 # set number of times each combination must be repeated

## Create vectors for parameters to vary across the simulated samples

par.sample.size <- c(2000) #(k1)

# parameter controlling the sample size

par.mn.tet1s <- c(-1, 0, 1) # mean theta T1 (k2)

# mean theta score at T1

par.sd.tet1s <- c(1) # SD theta T1 (k3)

# parameter controlling the SD of theta T1;

par.mn.imic <- c(0.5) # mean iMIC (theta change) (k4)

# parameter controlling the mean iMIC (= genuine MIC; gMIC);

par.sd.imic <- c(0.075) # SD iMIC (theta change) (k5)

# parameter controlling the SD of the iMICs

# TAKE CARE: some iMIC values should not approach zero as this

# will not be realistic for a 'minimal important' improvement

par.sd.tetchs <- c(0.75, 1, 1.25) # SD theta change (k6)

# parameter controlling the SD of theta change (T2-T1);

par.cor.t1.ch <- c(-0.5, 0) # cor between theta T1 and change (k7)

# parameter controlling the correlation between theta T1 the theta change;

# values represent correlation coefficients

par.cor.t1.imic <- c(0) # cor between theta T1 and iMIC (k8)

# parameter controlling the correlation between theta T1 and the iMICs;

# value represents a correlation coefficient

par.prop.imp <- c(0.2, 0.5, 0.8) # 'true' proportion improved (k9)

# parameter controlling the 'true' proportion improved, that is

# the proportion patients whose latent change exceed their iMIC.

par.rel.trt <- c(0.3, 0.5) # reliability of transition rating (k10)

# parameter controlling the reliability of the transiton rating (TRT).

# Actually the parameter controls the reliability of the "perceived change"

# relative to the true change.

par.psb <- c(0, 0.2, 0.4, 0.6, 0.8, 1) # PSB (k11)

# parameter controlling the present state bias of the transition

# rating (TRT); values represent the average proportion of present state

# that is included on the "weighted change"

## Calculate number of combinations and total number of simulations

npc <- length(par.sample.size) * length(par.mn.tet1s) * length(par.sd.tet1s) *

length(par.mn.imic) * length(par.sd.imic) * length(par.sd.tetchs) *

length(par.cor.t1.ch) * length(par.cor.t1.imic) * length(par.prop.imp) *

length(par.rel.trt) * length(par.psb)

npc # total number of combinations

nps <- npc * nr

nps # total number of simulated samples

index <- 0

## Create starting point for dataframe �dfp� to hold results of the simulations

sample.size.par <- as.numeric(rep(NA, nps))

mn.tet1s.par <- as.numeric(rep(NA, nps))

dfp <- data.frame(sample.size.par, mn.tet1s.par) # creates dataframe "dfp"

## create the other variables in "dfp"

dfp$sd.tet1s.par <- as.numeric(rep(NA, nps))

dfp$mn.imic.par <- as.numeric(rep(NA, nps))

dfp$sd.imic.par <- as.numeric(rep(NA, nps))

dfp$sd.tetchs.par <- as.numeric(rep(NA, nps))

dfp$cor.t1.ch.par <- as.numeric(rep(NA, nps))

dfp$cor.t1.imic.par <- as.numeric(rep(NA, nps))

dfp$prop.imp.par <- as.numeric(rep(NA, nps))

dfp$rel.trt.par <- as.numeric(rep(NA, nps))

dfp$psb.par <- as.numeric(rep(NA, nps))

dfp$prop.imp.trt <- as.numeric(rep(NA, nps)) # prop. improved based on TRT

dfp$prop.imp.tru <- as.numeric(rep(NA, nps)) # prop. imp. based on true change

dfp$mn.xo1 <- as.numeric(rep(NA, nps)) # mean final HRQOL score at T1

dfp$sd.xo1 <- as.numeric(rep(NA, nps)) # SD final HRQOL score at T1

dfp$skew.xo1 <- as.numeric(rep(NA, nps))

dfp$kurt.xo1 <- as.numeric(rep(NA, nps))

dfp$flor.xo1 <- as.numeric(rep(NA, nps))

dfp$ceil.xo1 <- as.numeric(rep(NA, nps))

dfp$mn.xo2 <- as.numeric(rep(NA, nps)) # mean final HRQOL score at T2

dfp$sd.xo2 <- as.numeric(rep(NA, nps)) # SD final HRQOL score at T2

dfp$skew.xo2 <- as.numeric(rep(NA, nps))

dfp$kurt.xo2 <- as.numeric(rep(NA, nps))

dfp$flor.xo2 <- as.numeric(rep(NA, nps))

dfp$ceil.xo2 <- as.numeric(rep(NA, nps))

dfp$mn.xoc <- as.numeric(rep(NA, nps)) # mean change score all subjects

dfp$sd.xoc <- as.numeric(rep(NA, nps)) # SD change score of all subjects

dfp$skew.xoc <- as.numeric(rep(NA, nps))

dfp$kurt.xoc <- as.numeric(rep(NA, nps))

dfp$flor.xoc <- as.numeric(rep(NA, nps))

dfp$ceil.xoc <- as.numeric(rep(NA, nps))

dfp$rel.xo1 <- as.numeric(rep(NA, nps)) # reliability observed score T1

dfp$mn.imic <- as.numeric(rep(NA, nps)) # mean iMIC (= gMIC)

dfp$sd.imic <- as.numeric(rep(NA, nps)) # SD of iMIC

dfp$cor.xoc.trt <- as.numeric(rep(NA, nps)) # correlation between TRT and observed HRQOL change score

dfp$pcor.xoc.trt <- as.numeric(rep(NA, nps)) # BISERIAL correlation between TRT and observed HRQOL change score

dfp$f1.cfa.uncon <- as.numeric(rep(NA, nps)) # Factor loading TRT on F1

dfp$f2.cfa.uncon <- as.numeric(rep(NA, nps)) # Factor loading TRT on F2

dfp$mean.f1.uncon <- as.numeric(rep(NA, nps)) # latent mean F1

dfp$mean.f2.uncon <- as.numeric(rep(NA, nps)) # latent mean F2

dfp$var.f1.uncon <- as.numeric(rep(NA, nps)) # latent variance F1

dfp$var.f2.uncon <- as.numeric(rep(NA, nps)) # latent variance F2

dfp$cov.f1f2.uncon <- as.numeric(rep(NA, nps)) # latent covariance F1-F2

dfp$f1.cfa.con <- as.numeric(rep(NA, nps)) # Factor loading TRT on F1

dfp$f2.cfa.con <- as.numeric(rep(NA, nps)) # Factor loading TRT on F2

dfp$psb.cfa <- as.numeric(rep(NA, nps)) # PSB estimated CFA model

dfp$mic.theta.cfa.uncon <- as.numeric(rep(NA, nps)) # MIC CFA unconstr. theta metric

dfp$mic.cfa.uncon <- as.numeric(rep(NA, nps)) # MIC CFA ETS unconstrained

dfp$mic.theta.cfa.con <- as.numeric(rep(NA, nps)) # MIC CFA ETS constr. theta metric

dfp$mic.cfa.con <- as.numeric(rep(NA, nps)) # MIC CFA ETS constrained

dfp$conv.con <- logical(nps) # Check model convergence

dfp$conv.uncon <- logical(nps) # Check model convergence

dfp$rel.uncon.trt <- as.numeric(rep(NA, nps)) # Reliability of TRs

dfp$rel.con.trt <- as.numeric(rep(NA, nps)) # Reliability of TRs

dfp$mic.mean <- as.numeric(rep(NA, nps)) # Mean change MIC

dfp$mic.roc <- as.numeric(rep(NA, nps)) # MIC ROC (youden)

dfp$mic.pred <- as.numeric(rep(NA, nps)) # MIC predictive

dfp$mic.adj <- as.numeric(rep(NA, nps)) # MIC adjusted

dfp$mic.true <- as.numeric(rep(NA, nps)) # MIC (ETS) true

dfp$f1.irt.uncon <- as.numeric(rep(NA, nps)) # Slope TRT on F1 unconstr.

dfp$f2.irt.uncon <- as.numeric(rep(NA, nps)) # Slope TRT on F2 unconstr.

dfp$f1.irt.con <- as.numeric(rep(NA, nps)) # Slope TRT on F1 constr.

dfp$f2.irt.con <- as.numeric(rep(NA, nps)) # Slope TRT on F1 constr.

dfp$mic.theta.irt.uncon <- as.numeric(rep(NA, nps)) # MIC IRT unconstr. theta metric

dfp$mic.irt.uncon <- as.numeric(rep(NA, nps)) # MIC IRT ETS unconstrained

dfp$mic.theta.irt.con <- as.numeric(rep(NA, nps)) # MIC IRT constr. theta metric

dfp$mic.irt.con <- as.numeric(rep(NA, nps)) # MIC IRT ETS constrained

dfp$psb.irt <- as.numeric(rep(NA, nps)) # PSB estimated IRT model

#############################################################################

### SIMULATIONS USING 'MIRT'

start.time <- Sys.time()

## --------------------------------

## Create repetions and perform the analyses

for(k1 in 1:length(par.sample.size)) {

for(k2 in 1:length(par.mn.tet1s)) {

for(k3 in 1:length(par.sd.tet1s)) {

for(k4 in 1:length(par.mn.imic)) {

for(k5 in 1:length(par.sd.imic)) {

for(k6 in 1:length(par.sd.tetchs)) {

for(k7 in 1:length(par.cor.t1.ch)) {

for(k8 in 1:length(par.cor.t1.imic)) {

for(k9 in 1:length(par.prop.imp)) {

for(k10 in 1:length(par.rel.trt)) {

for(k11 in 1:length(par.psb)) {

for(k12 in 1:nr) {

index <- (k1-1)*length(par.mn.tet1s)*length(par.sd.tet1s)*

length(par.mn.imic)*length(par.sd.imic)*length(par.sd.tetchs)*

length(par.cor.t1.ch)*length(par.cor.t1.imic)*length(par.prop.imp)*

length(par.rel.trt)*length(par.psb)*nr +

(k2-1)*length(par.sd.tet1s)*

length(par.mn.imic)*length(par.sd.imic)*length(par.sd.tetchs)*

length(par.cor.t1.ch)*length(par.cor.t1.imic)*length(par.prop.imp)*

length(par.rel.trt)*length(par.psb)*nr +

(k3-1)*length(par.mn.imic)*length(par.sd.imic)*length(par.sd.tetchs)*

length(par.cor.t1.ch)*length(par.cor.t1.imic)*length(par.prop.imp)*

length(par.rel.trt)*length(par.psb)*nr +

(k4-1)*length(par.sd.imic)*length(par.sd.tetchs)*

length(par.cor.t1.ch)*length(par.cor.t1.imic)*length(par.prop.imp)*

length(par.rel.trt)*length(par.psb)*nr +

(k5-1)*length(par.sd.tetchs)*

length(par.cor.t1.ch)*length(par.cor.t1.imic)*length(par.prop.imp)*

length(par.rel.trt)*length(par.psb)*nr +

(k6-1)*length(par.cor.t1.ch)*length(par.cor.t1.imic)*length(par.prop.imp)*

length(par.rel.trt)*length(par.psb)*nr +

(k7-1)*length(par.cor.t1.imic)*length(par.prop.imp)*

length(par.rel.trt)*length(par.psb)*nr +

(k8-1)*length(par.prop.imp)*

length(par.rel.trt)*length(par.psb)*nr +

(k9-1)*length(par.rel.trt)*length(par.psb)*nr +

(k10-1)*length(par.psb)*nr +

(k11-1)*nr + k12

print(index)

###############################################################

( dfp$sample.size.par[index] <- N <- par.sample.size[k1] )

( dfp$mn.tet1s.par[index] <- par.mn.tet1s[k2] )

( dfp$sd.tet1s.par[index] <- par.sd.tet1s[k3] )

( dfp$mn.imic.par[index] <- par.mn.imic[k4] )

( dfp$sd.imic.par[index] <- par.sd.imic[k5] )

( dfp$sd.tetchs.par[index] <- par.sd.tetchs[k6] )

( dfp$cor.t1.ch.par[index] <- par.cor.t1.ch[k7] )

( dfp$cor.t1.imic.par[index] <- par.cor.t1.imic[k8] )

( dfp$prop.imp.par[index] <- par.prop.imp[k9] )

( dfp$rel.trt.par[index] <- par.rel.trt[k10] )

( dfp$psb.par[index] <- par.psb[k11] )

### Create theta T1 and dataset 1 (baseline)

( rt1ch <- par.cor.t1.ch[k7] ) # correlation between tet1s and tetchs

Sigma <- matrix(c(1,rt1ch,rt1ch,1),2,2)

Sigma

tets <- mvrnorm(N, rep(0, 2), Sigma)

mean(tets[,1])

mean(tets[,2])

sd(tets[,1])

sd(tets[,2])

cor(tets[,1],tets[,2])

tet1s <- tets[,1]*par.sd.tet1s[k3]/sd(tets[,1]) # adjust SD

tet1s <- tet1s-mean(tet1s)+par.mn.tet1s[k2] # adjust mean

tet1s <- as.matrix(tet1s)

mean(tet1s)

sd(tet1s)

skewness(tet1s, type=2)

e1071::kurtosis(tet1s, type=2)

set.seed( sample(10000:20000,1) )

dat1 <- simdata(a1, d1, N, itemtype="graded", Theta=tet1s)

dat1 <- as.data.frame(dat1)

xo1 <- rowSums(dat1) # This is the baseline PROM score

( dfp$mn.xo1[index] <- mean(xo1) )

( dfp$sd.xo1[index] <- sd(xo1) )

( dfp$skew.xo1[index] <- skewness(xo1, type=2) )

( dfp$kurt.xo1[index] <- e1071::kurtosis(xo1, type=2) )

( dfp$flor.xo1[index] <- table(xo1)[1] / length(xo1) ) # floor effect

( dfp$ceil.xo1[index] <- table(xo1)[length(table(xo1))] / length(xo1) )# ceiling effect

( dfp$rel.xo1[index] <- psych::alpha(dat1)$total$raw_alpha ) # Cronbach's alpha XO1

### Create theta change

tetchs <- tets[,2]

cor(tet1s,tetchs)

tetchs <- tetchs - mean(tetchs) # make mean = 0

tetchs <- (tetchs/sd(tetchs))*par.sd.tetchs[k6] # transform SD of tetchs

( qtl <- quantile(tetchs, prob=(1-par.prop.imp[k9])) )

( mean.tetchs <- par.mn.imic[k4] - qtl ) # Estimate mean theta change to get

# the desired proportion improved

tetchs <- tetchs + mean.tetchs # transform mean of tetchs

mean(tetchs)

sd(tetchs)

skewness(tetchs, type=2)

e1071::kurtosis(tetchs, type=2)

### Create theta T2 and dataset 2 (follow-up)

tet2s <- as.matrix(tet1s + tetchs) # theta T2

mean(tet2s)

sd(tet2s)

skewness(tet2s, type=2)

kurtosis(tet2s)

cor(tet1s, tet2s)

set.seed( sample(30000:40000,1) )

dat2 <- simdata(a1, d1, N, itemtype="graded", Theta=tet2s)

dat2 <- as.data.frame(dat2)

xo2 <- rowSums(dat2) # This is the follow-up test (sum) score

( dfp$mn.xo2[index] <- mean(xo2) )

( dfp$sd.xo2[index] <- sd(xo2) )

( dfp$skew.xo2[index] <- skewness(xo2, type=2) )

( dfp$kurt.xo2[index] <- kurtosis(xo2)[1] )

( dfp$flor.xo2[index] <- table(xo2)[1] / length(xo2) )

( dfp$ceil.xo2[index] <- table(xo2)[length(table(xo2))] / length(xo2) )

### Create observed change score

xoc <- xo2 - xo1

( dfp$mn.xoc[index] <- mean(xoc) )

( dfp$sd.xoc[index] <- sd(xoc) )

( dfp$skew.xoc[index] <- skewness(xoc, type=2) )

( dfp$kurt.xoc[index] <- kurtosis(xoc)[1] )

( dfp$flor.xoc[index] <- table(xoc)[1] / length(xoc) )

( dfp$ceil.xoc[index] <- table(xoc)[length(table(xoc))] / length(xoc) )

#############################################################################

### CREATE PERCEIVED CHANGE WITH PRESENT STATE BIAS

( mn.psb <- par.psb[k11] )

( sd.psb <- (0.5 - abs(0.5 - par.psb[k11]))/4 )

psb <- rnorm(N,mn.psb,sd.psb)

psb[psb<0] <- 0

psb[psb>1] <- 1

min(psb)

max(psb)

# Weighted theta change is composed of a proportion (psb) of theta T2 - mean T1

# and a proportion (1-psb) of theta change.

# The mean of theta T1 is subtracted from theta T2.

# This way of weighting ensures that the weighted change retains the same

# (theta) metric as theta T1 and theta T2

# However, weighting can impact the sample proportion improved

tetch.wgt <- psb*(tet2s - mean(tet1s)) + (1-psb)*tetchs # weighted change

mean(tetchs)

sd(tetchs)

mean(tetch.wgt)

sd(tetch.wgt)

# Add ERROR to the weighted change: PERCEIVED CHANGE

( rel.trt <- par.rel.trt[k10] ) # reliability of the TRT

( sd.ch.error <- sqrt(((1-rel.trt)/rel.trt)*sd(tetch.wgt)^2) )

tetch.error <- rnorm(N, 0, sd.ch.error)

tetch.prc <- tetch.wgt + tetch.error

mean(tetch.wgt)

sd(tetch.wgt)

mean(tetch.prc)

sd(tetch.prc)

var(tetch.wgt)/var(tetch.prc) # reliability of the perceived change

cor(tetch.wgt,tetch.prc)^2 # reliability of the perceived change

# Create iMIC distribution and other tresholds

imic <- rnorm(N, par.mn.imic[k4], par.sd.imic[k5])

( dfp$mn.imic[index] <- mean(imic) )

( dfp$sd.imic[index] <- sd(imic) )

thrd1 <- rnorm(N, -1.5, par.sd.imic[k5])

thrd2 <- rnorm(N, -0.5, par.sd.imic[k5])

thrd3 <- imic

thrd4 <- rnorm(N, 1.5, par.sd.imic[k5])

# create TRANSITION RATINGS -- DICHOTOMOUS

trt <- numeric(N)

trt[tetch.prc > imic] <- 1

table(trt)

# create TRANSITION RATINGS --- POLYTOMOUS

trtp <- numeric(N)

trtp[tetch.prc > thrd1] <- 1

trtp[tetch.prc > thrd2] <- 2

trtp[tetch.prc > thrd3] <- 3

trtp[tetch.prc > thrd4] <- 4

table(trtp)

# TR-change correlations

( dfp$cor.xoc.trt[index] <- cor.trt.xoc <- cor(xoc,trt) ) # point-biserial

( dfp$pcor.xoc.trt[index] <- psych::biserial(xoc,trt) ) # biserial

# proportion truly improved based on true change

trt.tru <- numeric(N)

trt.tru[tetchs > imic] <- 1

table(trt.tru)

( dfp$prop.imp.tru[index] <- mean(trt.tru) )

# proportion improved based on perceived change

( dfp$prop.imp.trt[index] <- q <- mean(trt) )

( p <- log(q/(1-q)) ) # p = logodds(pre)

##############################################################

### True MIC, as simulated

cap <- capture.output( mod1 <- mirt(dat1, itemtype='graded') )

# MIC.ets based on baseline distribution

theta1 <- as.matrix( rnorm(500000,0,1) )

theta2 <- theta1 + par.mn.imic[k4]

( mean.ets1 <- mean(expected.test(mod1, theta1)) )

( mean.ets2 <- mean(expected.test(mod1, theta2)) )

( dfp$mic.true[index] <- mean.ets2 - mean.ets1 )

### Mean change MIC

mean(xoc[trtp==0])

mean(xoc[trtp==1])

mean(xoc[trtp==2]) # "unchanged" category

( dfp$mic.mean[index] <- mean(xoc[trtp==3]) ) # "little better" category

mean(xoc[trtp==4])

### ROC analysis

rocobj <- roc(trt, xoc, quiet = TRUE)

( mic.roc <- coords(rocobj, x="best", input="threshold", ret="threshold",

best.method="youden", transpose = TRUE) )

( dfp$mic.roc[index] <- mic.roc[sample(length(mic.roc),1)] )

### LCFA METHOD USING LAVAAN

datw <- data.frame(dat1,dat2,trt)

## Collapse categories with less than 1 response (if necessary)

## Same items need to have the same number of response options at T1 and T2

nitems = 10

noptions = 4

mat <- matrix(rep(NA,noptions*2),ncol=2)

colnames(mat) <- c("T1","T2")

rownames(mat) <- c("0","1","2","3")

for(k in 1:nitems) {

hlp <- datw[,c(k, nitems+k)]

for(j in 1:2) {

for(i in 0:3) {

mat[i+1,j] <- cnt <- length(hlp[,j][hlp[,j]==i])

if(cnt<1 & i==0) {

datw[,k][datw[,k]==i] <- 1

datw[,nitems+k][datw[,nitems+k]==i] <- 1

}

if(cnt<1 & i==3) {

datw[,k][datw[,k]==i] <- 2

datw[,nitems+k][datw[,nitems+k]==i] <- 2

}

}

}

}

for(k in 1:nitems) {

hlp <- datw[,c(k, nitems+k)]

for(j in 1:2) {

for(i in 0:3) {

mat[i+1,j] <- cnt <- length(hlp[,j][hlp[,j]==i])

if(cnt<1 & i==1) {

datw[,k][datw[,k] <= i] <- 2

datw[,nitems+k][datw[,nitems+k] <= i] <- 2

}

if(cnt<1 & i==2) {

datw[,k][datw[,k] >= i] <- 1

datw[,nitems+k][datw[,nitems+k] >= i] <- 1

}

}

}

}

### Measurement equivalence model

mod.cat <- '

FU1 =~ Item_1+Item_2+Item_3+Item_4+Item_5+Item_6+

Item_7+Item_8+Item_9+Item_10

FU2 =~ Item_1.1+Item_2.1+Item_3.1+Item_4.1+Item_5.1+

Item_6.1+Item_7.1+Item_8.1+Item_9.1+Item_10.1

'

## the 2 factors are actually the same factor (FU) measured twice

longFacNames <- list(FU = c("FU1","FU2"))

## scalar invariance

syntax.scalar <- measEq.syntax(configural.model = mod.cat, data = datw,

ordered = T,

parameterization = "theta",

ID.fac = "std.lv", ID.cat = "Wu.Estabrook.2016",

longFacNames = longFacNames,

long.equal = c("thresholds","loadings",

"intercepts"))

# summary(syntax.scalar) # summarize model features

mod.scalar <- as.character(syntax.scalar) # save as text

# cat(mod.scalar) # print/view lavaan syntax

model.addition <- '

FU1 =~ NA*trt + f1*trt

FU2 =~ NA*trt + f2*trt

trt | NA*t1 + thr.trt*t1

mic := thr.trt/f2

psb := f1/f2+1

'

model <- paste0(mod.scalar,model.addition)

try( fit <- cfa(model, data=datw, ordered=T,

parameterization="theta") , silent=T )

if(fit@optim$converged==TRUE) {

( dfp$conv.uncon[index] <- TRUE )

pe <- parameterEstimates(fit, rsquare=T)

( dfp$rel.uncon.trt[index] <- rel.trt <- pe$est[pe$lhs=="trt" & pe$op=="r2"] ) # R-squared TRT

( dfp$f1.cfa.uncon[index] <- pe$est[pe$label=="f1"] ) # loading TRT on F1

( dfp$f2.cfa.uncon[index] <- pe$est[pe$label=="f2"] ) # loading TRT on F2

( dfp$mean.f1.uncon[index] <- pe$est[pe$lhs=="FU1" & pe$op=="~1"] )

( dfp$mean.f2.uncon[index] <- pe$est[pe$lhs=="FU2" & pe$op=="~1"] )

( dfp$var.f1.uncon[index] <- pe$est[pe$lhs=="FU1" & pe$rhs=="FU1"] )

( dfp$var.f2.uncon[index] <- pe$est[pe$lhs=="FU2" & pe$rhs=="FU2"] )

( dfp$cov.f1f2.uncon[index] <- pe$est[pe$lhs=="FU1" & pe$rhs=="FU2"] )

( dfp$mic.theta.cfa.uncon[index] <- mic.theta <- pe$est[pe$label=="mic"] )

( dfp$psb.cfa[index] <- pe$est[pe$label=="psb"] )

# MIC ETS

# MIC.ets based on baseline distribution

theta1 <- as.matrix( rnorm(500000,0,1) )

theta2 <- theta1 + mic.theta

( mean.ets1 <- mean(expected.test(mod1, theta1)) )

( mean.ets2 <- mean(expected.test(mod1, theta2)) )

( dfp$mic.cfa.uncon[index] <- mean.ets2 - mean.ets1 )

rm(mic.theta)

rm(pe)

rm(fit)

}

### Constrained LCFA model

model.addition <- '

f1 == -f2

f2 > 0 # positive constraint on f2

'

model.con <- paste0(model,model.addition)

try( fit <- cfa(model.con, data=datw, ordered=T,

parameterization="theta") , silent=T )

if(fit@optim$converged==TRUE) {

( dfp$conv.con[index] <- TRUE )

pe <- parameterEstimates(fit, rsquare=T)

( dfp$rel.con.trt[index] <- pe$est[pe$lhs=="trt" & pe$op=="r2"] ) # R-squared TRT

( dfp$f1.cfa.con[index] <- pe$est[pe$label=="f1"] ) # loading TRT on F1

( dfp$f2.cfa.con[index] <- pe$est[pe$label=="f2"] ) # loading TRT on F2

( dfp$mic.theta.cfa.con[index] <- mic.theta <- pe$est[pe$label=="mic"] )

# MIC ETS

# MIC.ets based on baseline distribution

# theta1 <- as.matrix( rnorm(500000,0,1) )

theta2 <- theta1 + mic.theta

# ( mean.ets1 <- mean(expected.test(mod1, theta1)) )

( mean.ets2 <- mean(expected.test(mod1, theta2)) )

( dfp$mic.cfa.con[index] <- mean.ets2 - mean.ets1 )

rm(mic.theta)

rm(pe)

rm(fit)

}

### LIRT model without accounting for local dependence, UNCONSTRAINED

# Accounting for local dependence requires modeling specific factors for

# each item across time, this is very computationally intense.

# However, as we did not simulate local item dependence, we refrained from

# taking any LD into account.

mod.irt <- 'Time1 = 1-10, 21

Time2 = 11-20, 21

COV = Time2*Time2, Time1*Time2 # Variance Time2 and covariance T1-T2 free

MEAN = Time2'

nitems = 10

# construct constraints dynatically

# obtain starting values

sv <- mirt(datw, mod.irt, pars='values')

# sv[,1:9]

# set up within time constraints

wtconstr <- sv$parnum[(sv$name == 'a1' | sv$name == 'a2') & sv$est]

# wtconstr

# create constraint list

constraints <- list()

itemnames <- colnames(datw)

pick <- c(0, nitems)

for(i in 1:nitems){

# accross time item constraints

# constraints[[paste0('slope.', i)]] <- sv$parnum[sv$name == paste0('a',2+i) & sv$est]

for(j in 1:(noptions-1)){

constraints[[paste0('intercept.', i, '_', j)]] <-

sv$parnum[sv$name == paste0('d',j) & (sv$item %in% itemnames[pick + i]) & sv$est]

}

# across time constraints

constraints[[paste0('Time.', i)]] <- wtconstr[pick + i]

}

cap <- capture.output( moda <- mirt(datw, mod.irt, constrain=constraints,

TOL=1e-3, itemtype = 'graded', optimizer = 'nlminb') )

# MIC in terms of theta change

( cf <- coef(moda, simplify=TRUE)$items ) # Variance-covariance matrix

( dfp$mic.theta.irt.uncon[index] <- mic.theta <- -cf[2*nitems+1,3]/cf[2*nitems+1,2] )

( dfp$f1.irt.uncon[index] <- cf[2*nitems+1,1] ) # slope TRT on F1

( dfp$f2.irt.uncon[index] <- cf[2*nitems+1,2] ) # slope TRT on F2

( dfp$psb.irt[index] <- cf[2*nitems+1,1]/cf[2*nitems+1,2] + 1 )

# MIC ETS

# MIC.ets based on baseline distribution

# theta1 <- as.matrix( rnorm(500000,0,1) )

theta2 <- theta1 + mic.theta

# ( mean.ets1 <- mean(expected.test(mod1, theta1)) )

( mean.ets2 <- mean(expected.test(mod1, theta2)) )

( dfp$mic.irt.uncon[index] <- mean.ets2 - mean.ets1 )

rm(mic.theta)

rm(moda)

### IRT model without accounting for local dependence, CONSTRAINED

mod.irt <- 'Time1 = 1-10, 21

Time2 = 11-20, 21

COV = Time2*Time2, Time1*Time2 # Variance Time2 and covariance T1-T2 free

MEAN = Time2'

nitems = 10

noptions = 4

# construct constraints dynatically

# obtain starting values

sv <- mirt(datw, mod.irt, pars='values')

# sv[,1:9]

# set up within time constraints

wtconstr <- sv$parnum[(sv$name == 'a1' | sv$name == 'a2') & sv$est]

# wtconstr

# create constraint list

constraints <- list()

itemnames <- colnames(datw)

pick <- c(0, nitems)

for(i in 1:nitems){

# accross time item constraints

# constraints[[paste0('slope.', i)]] <- sv$parnum[sv$name == paste0('a',2+i) & sv$est]

for(j in 1:(noptions-1)){

constraints[[paste0('intercept.', i, '_', j)]] <-

sv$parnum[sv$name == paste0('d',j) & (sv$item %in% itemnames[pick + i]) & sv$est]

}

# across time constraints

constraints[[paste0('Time.', i)]] <- wtconstr[pick + i]

}

# equal-but-opposite constraint

( trt.pars <- sv$parnum[sv$item=="trt" & (sv$name=="a1" | sv$name=="a2")] )

nconstrain <- list(trt.pars)

technical <- list(nconstrain=nconstrain)

try( cap <- capture.output( moda <- mirt(datw, mod.irt, constrain=constraints, TOL=1e-3,

itemtype = 'graded', technical = technical, optimizer = 'nlminb') ), silent=T )

# MIC in terms of theta change

( cf <- coef(moda, simplify=TRUE)$items ) # Variance-covariance matrix

( dfp$mic.theta.irt.con[index] <- mic.theta <- -cf[2*nitems+1,3]/cf[2*nitems+1,2] )

( dfp$f1.irt.con[index] <- cf[2*nitems+1,1] ) # slope TRT on F1

( dfp$f2.irt.con[index] <- cf[2*nitems+1,2] ) # slope TRT on F2

# MIC ETS

# MIC.ets based on baseline distribution

# theta1 <- as.matrix( rnorm(500000,0,1) )

theta2 <- theta1 + mic.theta

# ( mean.ets1 <- mean(expected.test(mod1, theta1)) )

( mean.ets2 <- mean(expected.test(mod1, theta2)) )

( dfp$mic.irt.con[index] <- mean.ets2 - mean.ets1 )

rm(mic.theta)

rm(moda)

### Do logistic regression and calculate parameters and MIC(pred)

mylogit <- glm(trt ~ xoc, family = "binomial")

C <- coef(mylogit)[1] # intercept coefficient C

B <- coef(mylogit)[2] # regression coefficient B

( dfp$mic.pred[index] <- mic.pred <- (p-C)/B ) # MIC(predicted)

## Improved adjusted MIC

rf <- (0.8/rel.trt - 0.5) * sd(xoc) * cor.trt.xoc

( dfp$mic.adj[index] <- mic.pred - rf * p )

}

}

}

}

}

}

}

}

}

}

}

}

end.time <- Sys.time()

time.taken <- end.time - start.time

time.taken

beep(5)

write.table(dfp , file = "E:/Simulations-MIC-PSB-3240.txt",

sep = " ", row.names = F, col.names = T)

#############################################################################

#### ####

#### ANALYSIS ####

#### ####

#############################################################################

## Read in the simulated data

dfp <- read.table(file.choose(), header=T)

# read: "Simulations-MIC-PSB-3240.txt"

dim(dfp)

data.frame(names(dfp))

### ANALYSIS RESIDUALS

library(rsimsum)

## Create MIC residuals

dfp$mic.mean.res <- dfp$mic.mean - dfp$mic.true

dfp$mic.roc.res <- dfp$mic.roc - dfp$mic.true

dfp$mic.pred.res <- dfp$mic.pred - dfp$mic.true

dfp$mic.adj.res <- dfp$mic.adj - dfp$mic.true

dfp$mic.cfa.con.res <- dfp$mic.cfa.con - dfp$mic.true

dfp$mic.cfa.uncon.res <- dfp$mic.cfa.uncon - dfp$mic.true

dfp$mic.irt.con.res <- dfp$mic.irt.con - dfp$mic.true

dfp$mic.irt.uncon.res <- dfp$mic.irt.uncon - dfp$mic.true

### Sample characteristics, Table 2

round(summary(dfp$rel.xo1)[c(4,1,6)],2)

round(summary(dfp$mn.xo1)[c(4,1,6)],1)

round(summary(dfp$sd.xo1)[c(4,1,6)],1)

round(summary(dfp$skew.xo1)[c(4,1,6)],2)

round(summary(dfp$kurt.xo1)[c(4,1,6)],2)

round(summary(dfp$flor.xo1)[c(4,1,6)],2)

round(summary(dfp$ceil.xo1)[c(4,1,6)],2)

round(summary(dfp$mn.xo2)[c(4,1,6)],1)

round(summary(dfp$sd.xo2)[c(4,1,6)],1)

round(summary(dfp$skew.xo2)[c(4,1,6)],2)

round(summary(dfp$kurt.xo2)[c(4,1,6)],2)

round(summary(dfp$flor.xo2)[c(4,1,6)],2)

round(summary(dfp$ceil.xo2)[c(4,1,6)],2)

round(summary(dfp$mn.xoc)[c(4,1,6)],1)

round(summary(dfp$sd.xoc)[c(4,1,6)],1)

round(summary(dfp$skew.xoc)[c(4,1,6)],2)

round(summary(dfp$kurt.xoc)[c(4,1,6)],2)

round(summary(dfp$flor.xoc)[c(4,1,6)],2)

round(summary(dfp$ceil.xoc)[c(4,1,6)],2)

# Fig. 1. Boxplots MIC residuals

ggplot(dfp,

aes(x=as.factor(psb.par), y=mic.mean.res)) +

geom_boxplot(notch=F) +

dev.new(width=5, height=4) +

scale_y_continuous(limits=c(-10, 10), breaks=c(-10,-5,0,5,10))

ggplot(dfp,

aes(x=as.factor(psb.par), y=mic.roc.res)) +

geom_boxplot(notch=F) +

dev.new(width=5, height=4) +

scale_y_continuous(limits=c(-10, 10), breaks=c(-10,-5,0,5,10))

ggplot(dfp,

aes(x=as.factor(psb.par), y=mic.pred.res)) +

geom_boxplot(notch=F) +

dev.new(width=5, height=4) +

scale_y_continuous(limits=c(-10, 10), breaks=c(-10,-5,0,5,10))

ggplot(dfp,

aes(x=as.factor(psb.par), y=mic.adj.res)) +

geom_boxplot(notch=F) +

dev.new(width=5, height=4) +

scale_y_continuous(limits=c(-10, 10), breaks=c(-10,-5,0,5,10))

ggplot(dfp,

aes(x=as.factor(psb.par), y=mic.irt.con.res)) +

geom_boxplot(notch=F) +

dev.new(width=5, height=4) +

scale_y_continuous(limits=c(-10, 10), breaks=c(-10,-5,0,5,10))

ggplot(dfp,

aes(x=as.factor(psb.par), y=mic.irt.uncon.res)) +

geom_boxplot(notch=F) +

dev.new(width=5, height=4) +

scale_y_continuous(limits=c(-10, 10), breaks=c(-10,-5,0,5,10))

ggplot(dfp,

aes(x=as.factor(psb.par), y=mic.cfa.con.res)) +

geom_boxplot(notch=F) +

dev.new(width=5, height=4) +

scale_y_continuous(limits=c(-10, 10), breaks=c(-10,-5,0,5,10))

ggplot(dfp,

aes(x=as.factor(psb.par), y=mic.cfa.uncon.res)) +

geom_boxplot(notch=F) +

dev.new(width=5, height=4) +

scale_y_continuous(limits=c(-10, 10), breaks=c(-10,-5,0,5,10))

## True MIC (in terms of PROM change score)

summary(dfp$mic.true)

sd(dfp$mic.true)

summary(dfp[dfp$mn.tet1s.par==0,]$mic.true)

sd(dfp[dfp$mn.tet1s.par==0,]$mic.true)

summary(dfp[dfp$mn.tet1s.par==-1,]$mic.true)

sd(dfp[dfp$mn.tet1s.par==-1,]$mic.true)

summary(dfp[dfp$mn.tet1s.par==1,]$mic.true)

sd(dfp[dfp$mn.tet1s.par==1,]$mic.true)

ggplot(dfp,

aes(x=as.factor(mn.tet1s.par), y=mic.true)) +

geom_boxplot(notch=F) +

dev.new(width=5, height=4) +

scale_y_continuous(limits=c(2, 4), breaks=c(2,2.5,3,3.5,4))

### Effect of PSB

## MIC Mean

mean(dfp[dfp$psb.par==0,]$mic.mean.res)

sd(dfp[dfp$psb.par==0,]$mic.mean.res)

s1 <- simsum(data = dfp, estvarname = "mic.mean.res", true = 0,

methodvar = "psb.par", ref = "0")

ss1 <- summary(s1)

ss1

## MIC ROC

s1 <- simsum(data = dfp, estvarname = "mic.roc.res", true = 0,

methodvar = "psb.par", ref = "0")

ss1 <- summary(s1)

ss1

## MIC pred

s1 <- simsum(data = dfp, estvarname = "mic.pred.res", true = 0,

methodvar = "psb.par", ref = "0")

ss1 <- summary(s1)

ss1

## MIC adjusted

s1 <- simsum(data = dfp, estvarname = "mic.adj.res", true = 0,

methodvar = "psb.par", ref = "0")

ss1 <- summary(s1)

ss1

## MIC LIRT constrained

s1 <- simsum(data = dfp, estvarname = "mic.irt.con.res", true = 0,

methodvar = "psb.par", ref = "0")

ss1 <- summary(s1)

ss1

## MIC LIRT UNconstrained

s1 <- simsum(data = dfp, estvarname = "mic.irt.uncon.res", true = 0,

methodvar = "psb.par", ref = "0")

ss1 <- summary(s1)

ss1

## MIC LCFA constrained

s1 <- simsum(data = dfp, estvarname = "mic.cfa.con.res", true = 0,

methodvar = "psb.par", ref = "0")

ss1 <- summary(s1)

ss1

## MIC LCFA UNconstrained

s1 <- simsum(data = dfp, estvarname = "mic.cfa.uncon.res", true = 0,

methodvar = "psb.par", ref = "0")

ss1 <- summary(s1)

ss1

#########################################################################

### REGRESSION ANALYSIS

## MIC mean

fit <- lm(mic.mean.res ~

mn.tet1s.par*

sd.tetchs.par*

cor.t1.ch.par*

prop.imp.par*

rel.trt.par*

psb.par,

data = dfp)

summary(fit)

# Multiple R-squared: 0.9799, Adjusted R-squared: 0.9795

# Intermediate steps not shown

# FINAL MODEL

fit <- lm(mic.mean.res ~

prop.imp.par+

mn.tet1s.par:prop.imp.par,

data = dfp)

summary(fit)

# Multiple R-squared: 0.9092, Adjusted R-squared: 0.9091

ggplot(dfp,

aes(x=as.factor(prop.imp.par), y=mic.mean.res, colour=factor(mn.tet1s.par))) +

geom_boxplot(notch=F) +

dev.new(width=6.6, height=4) +

scale_y_continuous(limits=c(-10, 10), breaks=c(-10,-5,0,5,10))

mean(dfp[dfp$prop.imp.par==0.5,]$mic.mean.res)

## MIC ROC

fit <- lm(mic.roc.res ~

mn.tet1s.par*

sd.tetchs.par*

cor.t1.ch.par*

prop.imp.par*

rel.trt.par*

psb.par,

data = dfp)

summary(fit)

# Multiple R-squared: 0.8188, Adjusted R-squared: 0.8152

# Intermediate steps not shown

## FINAL MODEL

fit <- lm(mic.roc.res ~

prop.imp.par+

mn.tet1s.par:prop.imp.par,

data = dfp)

summary(fit)

# Multiple R-squared: 0.741, Adjusted R-squared: 0.7409

ggplot(dfp,

aes(x=as.factor(prop.imp.par), y=mic.roc.res, colour=factor(mn.tet1s.par))) +

geom_boxplot(notch=F) +

dev.new(width=6.6, height=4) +

scale_y_continuous(limits=c(-10, 10), breaks=c(-10,-5,0,5,10))

#### MIC pred

fit <- lm(mic.pred.res ~

mn.tet1s.par*

sd.tetchs.par*

cor.t1.ch.par*

prop.imp.par*

rel.trt.par*

psb.par,

data = dfp)

summary(fit)

# Multiple R-squared: 0.9918, Adjusted R-squared: 0.9917

# Intermediate steps not shown

# FINAL MODEL:

fit <- lm(mic.pred.res ~

prop.imp.par,

data = dfp)

summary(fit)

# Multiple R-squared: 0.9202, Adjusted R-squared: 0.9202

ggplot(dfp,

aes(x=as.factor(prop.imp.par), y=mic.pred.res)) +

geom_boxplot(notch=F) +

dev.new(width=5, height=4) +

scale_y_continuous(limits=c(-10, 10), breaks=c(-10,-5,0,5,10))

#### MIC Adjusted

fit <- lm(mic.adj.res ~

mn.tet1s.par*

sd.tetchs.par*

cor.t1.ch.par*

prop.imp.par*

rel.trt.par*

psb.par,

data = dfp)

summary(fit)

# Multiple R-squared: 0.8884, Adjusted R-squared: 0.8862

# Intermediate steps not shown

## INTERMEDIATE MODEL

fit <- lm(mic.adj.res ~

mn.tet1s.par+

cor.t1.ch.par+

psb.par+

mn.tet1s.par:cor.t1.ch.par+

cor.t1.ch.par:prop.imp.par+

prop.imp.par:psb.par,

data = dfp)

summary(fit)

# Multiple R-squared: 0.841, Adjusted R-squared: 0.8407

ggplot(dfp,

aes(x=as.factor(psb.par), y=mic.adj.res, colour=factor(prop.imp.par))) +

geom_boxplot(notch=F) +

dev.new(width=6.6, height=4) +

scale_y_continuous(limits=c(-10, 10), breaks=c(-10,-5,0,5,10))

# FINAL MODEL + SQUARED PSB

fit <- lm(mic.adj.res ~

mn.tet1s.par+

I(psb.par^2)+

prop.imp.par:I(psb.par^2)+

mn.tet1s.par:cor.t1.ch.par,

data = dfp)

summary(fit)

# Multiple R-squared: 0.8229, Adjusted R-squared: 0.8227

#### MIC LIRT (constrained)

fit <- lm(mic.irt.con.res ~

mn.tet1s.par*

sd.tetchs.par*

cor.t1.ch.par*

prop.imp.par*

rel.trt.par*

psb.par,

data = dfp)

summary(fit)

# Multiple R-squared: 0.7443, Adjusted R-squared: 0.7392

# Intermediate steps not shown

## INTERMEDIATE MODEL:

fit <- lm(mic.irt.con.res ~

prop.imp.par+

cor.t1.ch.par:psb.par+

sd.tetchs.par:cor.t1.ch.par:psb.par+

cor.t1.ch.par:prop.imp.par:psb.par+

sd.tetchs.par:cor.t1.ch.par:prop.imp.par:psb.par,

data = dfp)

summary(fit)

# Multiple R-squared: 0.7084, Adjusted R-squared: 0.708

ggplot(dfp,

aes(x=as.factor(psb.par), y=mic.irt.con.res, colour=factor(cor.t1.ch.par))) +

geom_boxplot(notch=F) +

dev.new(width=6.6, height=4) +

scale_y_continuous(limits=c(-10, 10), breaks=c(-10,-5,0,5,10))

ggplot(dfp,

aes(x=as.factor(psb.par), y=mic.irt.con.res, colour=factor(prop.imp.par))) +

geom_boxplot(notch=F) +

dev.new(width=6.6, height=4) +

scale_y_continuous(limits=c(-10, 10), breaks=c(-10,-5,0,5,10))

## FINAL MODEL WITH PSB-SQUARED

# Intermediate steps not shown

fit <- lm(mic.irt.con.res ~

I(cor.t1.ch.par * psb.par^2)+

I(sd.tetchs.par*cor.t1.ch.par*psb.par^2)+

I(cor.t1.ch.par*prop.imp.par*psb.par^2)+

I(sd.tetchs.par*cor.t1.ch.par*prop.imp.par*psb.par^2),

data = dfp)

summary(fit)

# Multiple R-squared: 0.7147, Adjusted R-squared: 0.7144

#### MIC LIRT, UNconstrained

fit <- lm(mic.irt.uncon.res ~

mn.tet1s.par*

sd.tetchs.par*

cor.t1.ch.par*

prop.imp.par*

rel.trt.par*

psb.par,

data = dfp)

summary(fit)

# Multiple R-squared: 0.01927, Adjusted R-squared: -0.0001839

fit <- lm(mic.irt.uncon.res ~

mn.tet1s.par+

sd.tetchs.par+

cor.t1.ch.par+

prop.imp.par+

rel.trt.par+

psb.par,

data = dfp)

summary(fit)

# Multiple R-squared: 0.003068, Adjusted R-squared: 0.001218

#### MIC LCFA, constrained

fit <- lm(mic.cfa.con.res ~

mn.tet1s.par*

sd.tetchs.par*

cor.t1.ch.par*

prop.imp.par*

rel.trt.par*

psb.par,

data = dfp)

summary(fit)

# Multiple R-squared: 0.9064, Adjusted R-squared: 0.9045

# Intermediate steps not shown

## FINAL MODEL

fit <- lm(mic.cfa.con.res ~

psb.par+

mn.tet1s.par:psb.par+

prop.imp.par:psb.par+

sd.tetchs.par:cor.t1.ch.par:psb.par+

cor.t1.ch.par:prop.imp.par:psb.par+

sd.tetchs.par:cor.t1.ch.par:prop.imp.par:psb.par,

data = dfp)

summary(fit)

# Multiple R-squared: 0.834, Adjusted R-squared: 0.8337

ggplot(dfp,

aes(x=as.factor(psb.par), y=mic.cfa.con.res, colour=factor(cor.t1.ch.par))) +

geom_boxplot(notch=F) +

dev.new(width=6.6, height=4) +

scale_y_continuous(limits=c(-10, 10), breaks=c(-10,-5,0,5,10))

ggplot(dfp,

aes(x=as.factor(psb.par), y=mic.cfa.con.res, colour=factor(prop.imp.par))) +

geom_boxplot(notch=F) +

dev.new(width=6.6, height=4) +

scale_y_continuous(limits=c(-10, 10), breaks=c(-10,-5,0,5,10))

#### MIC LCFA, UNconstrained

fit <- lm(mic.cfa.uncon.res ~

mn.tet1s.par*

sd.tetchs.par*

cor.t1.ch.par*

prop.imp.par*

rel.trt.par*

psb.par,

data = dfp)

summary(fit)

# Multiple R-squared: 0.01975, Adjusted R-squared: 0.0003087

fit <- lm(mic.cfa.uncon.res ~

mn.tet1s.par+

sd.tetchs.par+

cor.t1.ch.par+

prop.imp.par+

rel.trt.par+

psb.par,

data = dfp)

summary(fit)

# Multiple R-squared: 0.003493, Adjusted R-squared: 0.001644

**6. R-code for the recommended methods**

#############################################################################

#### ####

#### ADJUSTED PREDICTIVE MODELING METHOD ####

#### ####

#############################################################################

## Acquire packages

library(mirt)

library(lavaan)

library(semTools)

library(MASS)

## Simulate some data

set.seed(12345)

b2 <- c(-0.8, -0.8, -0.4, -0.4, 0, 0, 0.4, 0.4, 0.8, 0.8)

bc <- b2/4

b1 <- b2 - 1 + sample(bc)

b3 <- b2 + 1 + sample(bc)

a1 <- sample( 1.7+b2/2 )

cf.simb <- as.matrix( data.frame(a1,b1,b2,b3) )

round(cf.simb, 3)

round(colMeans(cf.simb), 3)

cf.simb <- as.data.frame(cf.simb)

# Transform b-parameters to d-parameters ('mirt' works with d-parameters)

# difficulty (b) = easiness (d) / -a

cf.sim <- cf.simb

colnames(cf.sim) <- c("a1","d1","d2","d3")

cf.sim$d1 <- -cf.simb$b1*cf.sim$a1

cf.sim$d2 <- -cf.simb$b2*cf.sim$a1

cf.sim$d3 <- -cf.simb$b3*cf.sim$a1

a1 <- as.matrix(cf.sim[ , 1])

d1 <- as.matrix(cf.sim[ , -1])

# Define simulation parameters

par.sample.size <- N <- 2000

# parameter controlling the sample size

par.mn.tet1s <- 0

# mean theta score at T1

par.sd.tet1s <- 1

# parameter controlling the SD of theta T1

par.mn.imic <- 0.5

# parameter controlling the mean of the individual MICs (iMICs)

par.sd.imic <- 0.075

# parameter controlling the SD of the iMICs

par.sd.tetchs <- 1

# parameter controlling the SD of theta change (T2-T1)

par.cor.t1.ch <- -0.5

# parameter controlling the correlation between theta T1 the theta change

par.cor.t1.imic <- c(0)

# parameter controlling the correlation between theta T1 and the iMICs

par.prop.imp <- 0.2

# parameter controlling the 'true' proportion improved, that is

# the proportion patients whose latent change exceed their iMIC

par.rel.trt <- 0.5

# parameter controlling the reliability of the transiton rating (TRT)

par.psb <- 0.4

# parameter controlling the present state bias of the transition

# rating (TRT)

# Create theta T1 and dataset 1 (baseline)

( rt1ch <- par.cor.t1.ch )

Sigma <- matrix(c(1,rt1ch,rt1ch,1),2,2)

tets <- mvrnorm(N, rep(0, 2), Sigma)

cor(tets[,1],tets[,2])

tet1s <- tets[,1]*par.sd.tet1s/sd(tets[,1])

tet1s <- tet1s-mean(tet1s)+par.mn.tet1s

tet1s <- as.matrix(tet1s)

mean(tet1s)

sd(tet1s)

dat1 <- simdata(a1, d1, N, itemtype="graded", Theta=tet1s)

dat1 <- as.data.frame(dat1)

# Create theta change

tetchs <- tets[,2]

cor(tet1s,tetchs)

tetchs <- tetchs - mean(tetchs)

tetchs <- (tetchs/sd(tetchs))*par.sd.tetchs

( qtl <- quantile(tetchs, prob=(1-par.prop.imp)) )

( mean.tetchs <- par.mn.imic - qtl ) # Estimate mean theta change to get

# the desired proportion improved

tetchs <- tetchs + mean.tetchs # transform mean of tetchs

mean(tetchs)

sd(tetchs)

# Create theta T2 and dataset 2 (follow-up)

tet2s <- as.matrix(tet1s + tetchs)

mean(tet2s)

sd(tet2s)

cor(tet1s, tet2s)

# set.seed( sample(30000:40000,1) )

dat2 <- simdata(a1, d1, N, itemtype="graded", Theta=tet2s)

dat2 <- as.data.frame(dat2)

# Create perceived change with PSB

# Present state bias

( mn.psb <- par.psb )

( sd.psb <- (0.5 - abs(0.5 - par.psb))/4 )

psb <- rnorm(N,mn.psb,sd.psb)

psb[psb<0] <- 0

psb[psb>1] <- 1

min(psb)

max(psb)

# Weighted change

tetch.wgt <- psb*(tet2s - mean(tet1s)) + (1-psb)*tetchs

# Add ERROR to the weighted change: PERCEIVED CHANGE

# reliability of the TRT

( rel.trt <- par.rel.trt )

( sd.ch.error <- sqrt(((1-rel.trt)/rel.trt)*sd(tetch.wgt)^2) )

tetch.error <- rnorm(N, 0, sd.ch.error)

tetch.prc <- tetch.wgt + tetch.error

# Create iMIC distribution

imic <- rnorm(N, par.mn.imic, par.sd.imic)

# create TRANSITION RATINGS -- DICHOTOMOUS

trt <- numeric(N)

trt[tetch.prc > imic] <- 1

table(trt)

# DATASET

datw <- data.frame(dat1,dat2,trt)

names(datw)[1:10] <- paste0("v1_",1:10)

names(datw)[11:20] <- paste0("v2_",1:10)

# Dataset "datw" consists of 10 items for T1, 10 items for T2, and

# dichotomous transition ratings

### ADJUSTED PREDICTIVE MODELING

# Transition ratings reliability

model <- '

F1 =~ v1_1+v1_2+v1_3+v1_4+v1_5+v1_6+v1_7+v1_8+v1_9+v1_10+trt

F2 =~ v2_1+v2_2+v2_3+v2_4+v2_5+v2_6+v2_7+v2_8+v2_9+v2_10+trt

'

fit <- cfa(model, data=datw, ordered=T)

fitMeasures(fit, fit.measures = c("cfi.scaled", "tli.scaled", "rmsea.scaled",

"rmsea.ci.lower.scaled", "rmsea.ci.upper.scaled", "rmsea.pvalue.scaled",

"srmr"))

pe <- parameterEstimates(fit, rsquare=T)

( rel.TRT <- pe$est[pe$lhs=="trt" & pe$op=="r2"] )

# Calculate the baseline PROM score

xo1 <- rowSums(datw[,1:10])

# Calculate the follow-up PROM score

xo2 <- rowSums(datw[,11:20])

# Calculate the PROM change score

xoc <- xo2 - xo1

# proportion improved and logodds of improvement

trt <- datw$trt

( q <- mean(trt) ) # q = proportion improved

( p <- log(q/(1-q)) ) # p = logodds(pre)

# TR-change correlation

( cor.trt.xoc <- cor(xoc,trt) ) # point-biserial correlation

# Calculate predictive modeling MIC

mylogit <- glm(trt ~ xoc, family = "binomial")

C <- coef(mylogit)[1] # intercept coefficient C

B <- coef(mylogit)[2] # regression coefficient B

( mic.pred <- (p-C)/B ) # MIC(predicted)

# Calculate adjusted predictive modeling MIC

rf <- (0.8/rel.TRT - 0.5) * sd(xoc) * cor.trt.xoc

( mic.adj <- mic.pred - rf * p )

## Non-parametric bootstrapping for 95%-CI

ns <- 1000 # NR OF BOOTSTRAP SAMPLES

rel.Trating <- as.numeric(rep(NA,ns))

MIC.adjusted <- as.numeric(rep(NA,ns))

set.seed(1234)

for(m in 1:ns) {

print(m)

# Draw a bootstrap sample from datw

selection <- sample(1:dim(datw)[1], nrow(datw), replace=TRUE)

bootsamp <- datw[selection,]

# Transition ratings reliability

model <- '

F1 =~ v1_1+v1_2+v1_3+v1_4+v1_5+v1_6+v1_7+v1_8+v1_9+v1_10+trt

F2 =~ v2_1+v2_2+v2_3+v2_4+v2_5+v2_6+v2_7+v2_8+v2_9+v2_10+trt

'

fit <- cfa(model, data=bootsamp, ordered=T)

pe <- parameterEstimates(fit, rsquare=T)

( rel.Trating[m] <- pe$est[pe$lhs=="trt" & pe$op=="r2"] )

# Calculate the baseline PROM score

xo1 <- rowSums(bootsamp[,1:10])

# Calculate the follow-up PROM score

xo2 <- rowSums(bootsamp[,11:20])

# Calculate the PROM change score

xoc <- xo2 - xo1

# proportion improved and logodds of improvement

trt <- bootsamp$trt

( q <- mean(trt) ) # q = proportion improved

( p <- log(q/(1-q)) ) # p = logodds(pre)

# TR-change correlation

( cor.trt.xoc <- cor(xoc,trt) ) # point-biserial correlation

# Calculate predictive modeling MIC

mylogit <- glm(trt ~ xoc, family = "binomial")

C <- coef(mylogit)[1] # intercept coefficient C

B <- coef(mylogit)[2] # regression coefficient B

( mic.pred <- (p-C)/B ) # MIC(predicted)

# Calculate adjusted predictive modeling MIC

rf <- (0.8/rel.Trating[m] - 0.5) * sd(xoc) * cor.trt.xoc

( MIC.adjusted[m] <- mic.pred - rf * p )

}

# Results

mean(rel.Trating, na.rm=T) # mean TRT reliability

quantile(rel.Trating, c(0.025,0.975), na.rm=T) # 95% CI TRT reliability

mean(MIC.adjusted, na.rm=T) # mean Adjusted Predictive Modeling MIC

quantile(MIC.adjusted, c(0.025,0.975), na.rm=T) # 95% CI APM MIC

#############################################################################

#### ####

#### LIRT METHOD, UNCONSTRAINED ####

#### ####

#############################################################################

nitems = 10

noptions = 4

itemloadings <- rep(1:nitems, times = 2)

itemloadings <- c(itemloadings, NA)

model <- 'Time1 = 1-10, 21

Time2 = 11-20, 21

COV = Time2*Time2, Time1*Time2

MEAN = Time2'

# construct constraints dynamically

# obtain starting values

sv <- bfactor(datw, itemloadings, model, itemtype = 'graded', pars='values')

# set up within time constraints

wtconstr <- sv$parnum[(sv$name == 'a1' | sv$name == 'a2') & sv$est]

# create constraint list

constraints <- list()

itemnames <- colnames(datw)

pick <- c(0, nitems)

for(i in 1:nitems){

# accross time item constraints

constraints[[paste0('slope.', i)]] <- sv$parnum[sv$name == paste0('a',2+i) & sv$est]

for(j in 1:(noptions-1)){

constraints[[paste0('intercept.', i, '_', j)]] <-

sv$parnum[sv$name == paste0('d',j) & (sv$item %in% itemnames[pick + i]) & sv$est]

}

#across time constraints

constraints[[paste0('Time.', i)]] <- wtconstr[pick + i]

}

mod <- bfactor(datw, itemloadings, model, constrain=constraints, TOL=1e-3,

itemtype = 'graded', optimizer = 'nlminb')

## MIC in terms of theta change

cf <- coef(mod, simplify=TRUE)

( slope.TR1 <- cf$items[2*nitems+1,1] ) # slope TR1 parameter

( slope.TR2 <- cf$items[2*nitems+1,2] ) # slope TR2 parameter

( tr.t1 <- cf$items[2*nitems+1,nitems+3] ) # Intercept TR

# Present state bias

slope.TR1/slope.TR2 + 1

# LIRT-based MIC in terms of the theta metric

( mic.theta <- -tr.t1/slope.TR2 )

## MIC in terms of the PROM score

moda <- mirt(datw[,1:10])

# coef(moda, simplify=TRUE)

N.ets = 500000

theta1 <- as.matrix( rnorm(N.ets,0,1) )

theta2 <- theta1 + mic.theta

( mean.ets1 <- mean(expected.test(moda, theta1)) )

( mean.ets2 <- mean(expected.test(moda, theta2)) )

( MIC.ets <- mean.ets2 - mean.ets1 ) # MIC in terms of the PROM score

#############################################################################

#### ####

#### LCFA METHOD, UNCONSTRAINED ####

#### ####

#############################################################################

model <- '

# factors

F1 =~a11*v1_1+a12*v1_2+a13*v1_3+a14*v1_4+a15*v1_5+a16*v1_6+a17*v1_7+

a18*v1_8+a19*v1_9+a110*v1_10+f1*trt

F2 =~a21*v2_1+a22*v2_2+a23*v2_3+a24*v2_4+a25*v2_5+a26*v2_6+a27*v2_7+

a28*v2_8+a29*v2_9+a210*v2_10+f2*trt

# factor loading constraints

a11 == a21

a12 == a22

a13 == a23

a14 == a24

a15 == a25

a16 == a26

a17 == a27

a18 == a28

a19 == a29

a110 == a210

# Thresholds

v1_1 + v2_1 | b11*t1+b12*t2+b13*t3

v1_2 + v2_2 | b21*t1+b22*t2+b23*t3

v1_3 + v2_3 | b31*t1+b32*t2+b33*t3

v1_4 + v2_4 | b41*t1+b42*t2+b43*t3

v1_5 + v2_5 | b51*t1+b52*t2+b53*t3

v1_6 + v2_6 | b61*t1+b62*t2+b63*t3

v1_7 + v2_7 | b71*t1+b72*t2+b73*t3

v1_8 + v2_8 | b81*t1+b82*t2+b83*t3

v1_9 + v2_9 | b91*t1+b92*t2+b93*t3

v1_10 + v2_10 | b101*t1+b102*t2+b103*t3

# Correlated errors over time

v1_1~~v2_1

v1_2~~v2_2

v1_3~~v2_3

v1_4~~v2_4

v1_5~~v2_5

v1_6~~v2_6

v1_7~~v2_7

v1_8~~v2_8

v1_9~~v2_9

v1_10~~v2_10

# Variances/covariances

F1 ~~ 1*F1

F2 ~~ NA*F2

F1 ~~ NA*F2

v2_1 ~~ NA*v2_1

v2_2 ~~ NA*v2_2

v2_3 ~~ NA*v2_3

v2_4 ~~ NA*v2_4

v2_5 ~~ NA*v2_5

v2_6 ~~ NA*v2_6

v2_7 ~~ NA*v2_7

v2_8 ~~ NA*v2_8

v2_9 ~~ NA*v2_9

v2_10 ~~ NA*v2_10

# Means/intercepts

F1 ~ 0*1

F2 ~ NA*1

# Derived values

trt | thr.trt*t1

mic := thr.trt/f2

psb := f1/f2+1

trt ~~ r1*trt # Residual variance of trt

r1==1 # Constrained appropriate with theta parameterization

r2.trt := 1-r1 # trt reliability (in standardized solution).

'

fit <- cfa(model, data=datw, std.lv=T, ordered=T, parameterization="theta")

fitMeasures(fit, fit.measures = c("cfi.scaled", "tli.scaled", "rmsea.scaled",

"rmsea.ci.lower.scaled", "rmsea.ci.upper.scaled", "rmsea.pvalue.scaled",

"srmr"))

pe <- parameterEstimates(fit, rsquare=T)

( rel.trt <- pe$est[pe$lhs=="trt" & pe$op=="r2"] ) # Reliability TRT

( psb <- pe$est[pe$label=="psb"] ) # Present state bias

( MIC.theta <- pe$est[pe$label=="mic"] ) # MIC in terms of theta metric

## Parametric bootstrapping

# MIC and PSB

( micx <- monteCarloCI(fit, nRep=20000, fast=T, level=.95, plot=F)[1:2,] )

# Reliability TR

monteCarloCI(fit, nRep=20000, standardized = TRUE, fast=T,

level=.95, plot=F)[3,]

## MIC in terms of the PROM score

( mic.t95.low <- micx[1,2] )

( mic.t95.up <- micx[1,3] )

moda <- mirt(datw[,1:10])

# coef(moda, simplify=TRUE)

N.ets = 500000

theta1 <- as.matrix( rnorm(N.ets,0,1) )

theta2 <- theta1 + MIC.theta

mean.ets1 <- mean(expected.test(moda, theta1))

mean.ets2 <- mean(expected.test(moda, theta2))

( MIC.ets <- mean.ets2 - mean.ets1 ) # MIC in terms of the PROM score

# theta1 <- as.matrix( rnorm(N.ets,0,1) )

theta2 <- theta1 + mic.t95.low

mean.ets1 <- mean(expected.test(moda, theta1))

mean.ets2 <- mean(expected.test(moda, theta2))

( MIC.ci.lower <- mean.ets2 - mean.ets1 ) # MIC in terms of the PROM score

# theta1 <- as.matrix( rnorm(N.ets,0,1) )

theta2 <- theta1 + mic.t95.up

mean.ets1 <- mean(expected.test(moda, theta1))

mean.ets2 <- mean(expected.test(moda, theta2))

( MIC.ci.upper <- mean.ets2 - mean.ets1 ) # MIC in terms of the PROM score

##### LCFA-based MIC using the Measurement Equivalence syntax (semTools)

mod.cat <- '

F1 =~ v1_1+v1_2+v1_3+v1_4+v1_5+v1_6+v1_7+v1_8+v1_9+v1_10

F2 =~ v2_1+v2_2+v2_3+v2_4+v2_5+v2_6+v2_7+v2_8+v2_9+v2_10

'

# the 2 factors are actually the same factor (F) measured twice

longFacNames <- list(F = c("F1","F2"))

# scalar invariance

syntax.scalar <- measEq.syntax(configural.model = mod.cat, data = datw,

ordered = T,

parameterization = "theta",

ID.fac = "std.lv", ID.cat = "Wu.Estabrook.2016",

longFacNames = longFacNames,

long.equal = c("thresholds","loadings",

"intercepts"))

# summary(syntax.scalar) # summarize model features

mod.scalar <- as.character(syntax.scalar) # save as text

# cat(mod.scalar) # print/view lavaan syntax

model.addition <- '

F1 =~ f1*trt

F2 =~ f2*trt

trt | thr.trt*t1

mic := thr.trt/f2

psb := f1/f2+1

trt ~~ r1*trt # Residual variance of trt

r1==1 # Constrained appropriate with theta parameterization

r2.trt := 1-r1 # trt reliability (in standardized solution).

'

model <- paste0(mod.scalar,model.addition)

fit <- cfa(model, data=datw, std.lv=T, ordered=T, parameterization="theta")

fitMeasures(fit, fit.measures = c("cfi.scaled", "tli.scaled", "rmsea.scaled",

"rmsea.ci.lower.scaled", "rmsea.ci.upper.scaled", "rmsea.pvalue.scaled",

"srmr"))

pe <- parameterEstimates(fit, rsquare=T)

( rel.trt <- pe$est[pe$lhs=="trt" & pe$op=="r2"] ) # Reliability TRT

( psb <- pe$est[pe$label=="psb"] ) # Present state bias

( MIC.theta <- pe$est[pe$label=="mic"] ) # MIC in terms of theta metric

## Parametric bootstrapping

# MIC and PSB

( micx <- monteCarloCI(fit, nRep=20000, fast=T, level=.95, plot=F)[1:2,] )

# Reliability TR

monteCarloCI(fit, nRep=20000, standardized = TRUE, fast=T,

level=.95, plot=F)[3,]

## MIC in terms of the PROM score

( mic.t95.low <- micx[1,2] )

( mic.t95.up <- micx[1,3] )

moda <- mirt(datw[,1:10])

# coef(moda, simplify=TRUE)

N.ets = 500000

theta1 <- as.matrix( rnorm(N.ets,0,1) )

theta2 <- theta1 + MIC.theta

mean.ets1 <- mean(expected.test(moda, theta1))

mean.ets2 <- mean(expected.test(moda, theta2))

( MIC.ets <- mean.ets2 - mean.ets1 ) # MIC in terms of the PROM score

# theta1 <- as.matrix( rnorm(N.ets,0,1) )

theta2 <- theta1 + mic.t95.low

mean.ets1 <- mean(expected.test(moda, theta1))

mean.ets2 <- mean(expected.test(moda, theta2))

( MIC.ci.lower <- mean.ets2 - mean.ets1 ) # MIC in terms of the PROM score

# theta1 <- as.matrix( rnorm(N.ets,0,1) )

theta2 <- theta1 + mic.t95.up

mean.ets1 <- mean(expected.test(moda, theta1))

mean.ets2 <- mean(expected.test(moda, theta2))

( MIC.ci.upper <- mean.ets2 - mean.ets1 ) # MIC in terms of the PROM score

1. Taken from: Terluin B, Trigg A, Fromy P, Schuller W, Terwee CB, Bjorner JB. Estimating anchor-based minimal important change using longitudinal confirmatory factor analysis. Qual Life Res 2024; 33(4): 963-973, Supplement, Sect. 1 [↑](#footnote-ref-1)
